# Canonical and Non-canonical TGFβ Signaling Activate Autophagy in an ULK1-Dependent Manner

**DOI:** 10.3389/fcell.2021.712124

**Published:** 2021-10-25

**Authors:** Charles B. Trelford, Gianni M. Di Guglielmo

**Affiliations:** Department of Physiology and Pharmacology, Schulich School of Medicine and Dentistry, Western University, London, ON, Canada

**Keywords:** macroautophagy, ULK1, autophagic flux, mTOR, tumorigenesis, lung cancer, LC3B

## Abstract

The mechanism(s) in which transforming growth factor beta 1 (TGFβ) modulates autophagy in cancer remain unclear. Here, we characterized the TGFβ signaling pathways that induce autophagy in non-small cell lung cancer cells, using cells lines stably expressing GFP-LC3-RFP-LC3ΔG constructs that measure autophagic flux. We demonstrated that TGFβ1 increases Unc 51-like kinase 1 (ULK1) protein levels, 5′ adenosine monophosphate-activated protein kinase (AMPK)-dependent ULK1 phosphorylation at serine (S) 555 and ULK1 complex formation but decreases mechanistic target of rapamycin (mTOR) activity on ULK1. Further analysis revealed that the canonical Smad4 pathway and the non-canonical TGFβ activated kinase 1/tumor necrosis factor receptor-associated factor 6/P38 mitogen activated protein kinase (TAK1-TRAF6-P38 MAPK) pathway are important for TGFβ1-induced autophagy. The TAK1-TRAF6-P38 MAPK pathway was essential for downregulating mTOR S2448 phosphorylation, ULK1 S555 phosphorylation and autophagosome formation. Furthermore, although siRNA-mediated Smad4 silencing did not alter mTOR-dependent ULK1 S757 phosphorylation, it did reduce AMPK-dependent ULK1 S555 phosphorylation and autophagosome formation. Additionally, Smad4 silencing and inhibiting the TAK1-TRAF6-P38 MAPK pathway decreased autophagosome-lysosome co-localization in the presence of TGFβ. Our results suggest that the Smad4 and TAK1-TRAF6-P38 MAPK signaling pathways are essential for TGFβ-induced autophagy and provide specific targets for the inhibition of TGFβ in tumor cells that utilize autophagy in their epithelial-mesenchymal transition program.

## Introduction

Macroautophagy, hereafter referred to as autophagy, is a catabolic process facilitated by lysosomes and acidic late endosomes that degrade macromolecules and organelles to replenish the building blocks for nucleic acids, proteins, carbohydrates, and lipids (Feng et al., 2014). Virtually all cells increase the rate of autophagy (autophagic flux) to eliminate the influx of damaged cellular materials mediated by cell stress to survive (Ding et al., 2007). However, cells have mechanisms to dampen autophagic flux because excessive degradation may initiate cell death (Shi et al., 2012). For example, cells modulate autophagic flux through post-translational modifications of autophagy related protein 1 (ATG1) (Yang and Klionsky, 2020). The phosphorylation status and activation of ATG1—Unc 51-like kinase 1 (ULK1) in mammals—is determined by a balance between mechanistic target of rapamycin (mTOR) and 5′ adenosine monophosphate activated protein kinase (AMPK) activity (Hosokawa et al., 2009; Makhov et al., 2014). When the rate of autophagy is detrimental to cells, mTOR phosphorylates ULK1 at serine (S)757 to disrupt ULK1-AMPK interactions (Kim et al., 2011). Alternatively, cell stressors impede mTOR and activate AMPK to directly phosphorylate ULK1 at S317, S555 and S778 (Dorsey et al., 2009). AMPK-dependent phosphorylation of ULK1 results in the formation of the ULK1 complex (Zachari and Ganley, 2017).

Autophagic degradation requires multiple ATG proteins downstream of the ULK1 complex to generate double membrane vesicles known as autophagosomes that engulf cellular materials prior to fusing with lysosomes or late endosomes (Bernard and Klionsky, 2013). Briefly, the ULK1 complex initiates autophagy by phosphorylating beclin-1 at S30 to assemble a phosphoinositide-3 kinase (PI3K) complex (Russell et al., 2013; Park et al., 2018), which inserts phosphatidylinositol lipids into membranes to recruit ATG proteins responsible for autophagosome formation (Matsunaga et al., 2010). Autophagosome growth is facilitated via ATG12-ATG5-ATG16L1 complexes incorporating lipids and ATG8—microtubule-associated light-chain 3 (LC3) in mammals—into autophagosome membranes (Sakoh-Nakatogawa et al., 2013). Prior to membrane incorporation, LC3 is post-translationally modified into LC3-I and LC3-II, which involves an ATG4-dependent cleavage to expose a C-terminal glycine residue (LC3-I) that is conjugated to phosphatidylethanolamine (LC3-II) by ATG7 and ATG3 (Satoo et al., 2009). As autophagosomes develop, autophagy cargo receptors tether cellular materials destined for degradation to LC3-II (Dooley et al., 2014). Once autophagosomes fully form, they migrate via microtubules and kinesin toward lysosomes in perinuclear regions of cells (Cheng et al., 2016). Autophagosomes fuse with lysosomes to generate autophagolysosomes (Mackeh et al., 2013) that contain lysosomal enzymes responsible for degrading autophagosomes and their cellular cargo (Klionsky et al., 2014).

Although autophagy is important for cellular homeostasis and survival, the protective functions of autophagy act as a double-edged sword in tumorigenesis (Eskelinen, 2011; Moscat and Diaz-Meco, 2012). For example, autophagy has been linked to drug resistance (Zou et al., 2012), epithelial-mesenchymal transition (EMT) (Alizadeh et al., 2018), cell migration (Tuloup-Minguez et al., 2013), metastasis (Qin et al., 2015), anoikis resistance (Peng et al., 2013), and aggressive tumor phenotypes (Mathew et al., 2007). As such, there is a need to understand the signaling pathways that may activate autophagy to promote tumorigenesis. In the past decade, several reports have suggested that transforming growth factor beta (TGFβ) activates autophagy (Kiyono et al., 2009; Suzuki et al., 2010; Xu et al., 2012; Fu et al., 2014; Alizadeh et al., 2018; Trelford and Di Guglielmo, 2020). Interestingly, like autophagy, TGFβ signaling impedes tumor formation in normal cells, yet promotes metastatic potential in tumor cells (Katsuno et al., 2013). In particular, TGFβ ligands are upregulated in several tumor microenvironments to induce angiogenesis, EMT and compromise immune cell surveillance (Thomas and Massagué, 2005; Jung et al., 2017; Muppala et al., 2017).

TGFβ signaling is initiated when transforming growth factor beta receptor type III (TβRIII) presents TGFβ ligands to transforming growth factor beta receptor type II (TβRII). TβRII transphosphorylates the transforming growth factor beta receptor type I (TβRI) that phosphorylates receptor Smads (R-Smads) and non-Smad proteins (Gunaratne et al., 2014). In Smad-dependent (canonical) TGFβ signaling, once R-Smads (Smad2 and Smad3) are phosphorylated by TβRI, they are released from the Smad Anchor for Receptor Activation (SARA) proteins. R-Smads then enter the nucleus in the presence of the co-Smad, Smad4, where they regulate gene expression (Weiss and Attisano, 2013). In Smad-independent (non-canonical) TGFβ signaling, TβRI or TβRII phosphorylate non-Smad proteins such as TGFβ activated kinase 1 (TAK1), atypical protein kinase C (aPKC), Par6, and PI3K complexes that regulate several cellular processes such as apoptosis, migration, proliferation, adhesion, differentiation, post-translational modifications, transcription and motility (Zhang, 2009).

Depending on the cell type, some studies have suggested that TGFβ-dependent autophagy relies on Smad transcription factors to upregulate ATG genes (Kiyono et al., 2009) whereas others emphasize that TGFβ activates autophagy by impeding mTOR (Fu et al., 2014; Chang et al., 2020). However, the specific TGFβ signaling pathway responsible for autophagy remain(s) unclear. Furthermore, many studies investigating TGFβ-dependent autophagy relied on LC3 protein levels as a readout for autophagy, which provides an incomplete picture (Klionsky et al., 2016; Trelford and Di Guglielmo, 2020). For this reason, our previous work verified that TGFβ increased autophagic flux using cells stably expressing green fluorescent protein (GFP)-LC3-red fluorescent protein (RFP)-LC3ΔG (Trelford and Di Guglielmo, 2020). After ATG4 cleaves GFP-LC3-RFP-LC3ΔG to generate GFP-LC3 and RFP-LC3ΔG, RFP-LC3ΔG cannot be conjugated to a phosphatidylethanolamine nor be incorporated into the autophagosome membrane. Therefore, during autophagy, the GFP-LC3 is degraded whereas the RFP-LC3ΔG is resistant to autophagic degradation (Kaizuka et al., 2016). Here, using non-small cell lung cancer (NSCLC) cell lines expressing GFP-LC3-RFP-LC3ΔG, we evaluated the role of specific components of the TGFβ signaling pathway on autophagy. The purpose of this work was to identify TGFβ signaling pathways responsible for activating autophagy in NSCLC cell lines to highlight molecular targets for cancer therapy.

## Materials and Methods

### Antibodies and Reagents

Primary antibodies were purchased from the following vendors: anti-GAPDH (Cell Signalling Technology, 2118S), anti-phospho-S465/467-Smad2 (P-Smad2; Cell Signalling Technology, 3108L), anti-Smad2/3 (BD Transduction laboratories, 562586), anti-LC3B (Cell Signalling Technology, 9236S), anti-ULK1 (Cell Signalling Technology, 8054S), anti-phospho-S555-ULK1 (Cell Signalling Technology, 5869S), anti-phospho-S757-ULK1 (Cell Signalling Technology, 6888S), anti-ULK2 (Santa Cruz, sc-293453), anti-SARA (Cell Signalling Technology, 13285S), anti-Smad4 (Cell Signalling Technology, 38454S), anti-mTOR (Cell Signalling Technology, 2972S), anti-phospho-S2448-mTOR (Cell Signalling Technology, 2971S), anti-adenosine monophosphate-activated protein kinase α (AMPKα; Cell Signalling Technology, 2532S), anti-phospho-T172-AMPKα (P-AMPK; Cell Signalling Technology, 50081S), anti-aPKCζ (Santa Cruz, sc-17781), anti-aPKCι (Santa Cruz, sc-17837), anti-TAK1 (Cell Signalling Technology, 5206S), anti-TRAF6 (Cell Signalling Technology, 8028S), anti-cleaved PARP (Cell Signalling Technology, 5625S) and anti-TGFβRIII (Santa Cruz, sc-74511). Secondary antibodies used for western blot analysis were as follows: Horseradish-peroxidase (HRP)-conjugated goat anti-rabbit-IgG (Thermo Fisher Scientific, 31460) and goat anti-mouse-IgG (Thermo Fisher Scientific, 31430). Fluorescently conjugated donkey anti-rabbit or donkey anti-mouse antibodies (Life Technologies) were used for immunofluorescence studies. Hoechst stain (Invitrogen, H3569) was used to label nuclei prior to live cell imaging. The pharmacological agents used to inhibit signaling pathways were SB431542 (TGFβ receptors; Selleckchem, S1067), LY294002 (PI3K; Sigma Aldrich, L9908-1MG), P38 MAPK Inhibitor (Calbiochem, 506126), Compound C (AMPK; Calbiochem, 171260) and ULK-101 (ULK1 and ULK2; Sellechchem, S8793).

### siRNA Studies

si-Control (4457289) or two different human siRNA constructs were purchased from Thermo Fisher Scientific (silencer select) for each knockdown experiment. The siRNA targets were si-SARA (s17932 and s17933), si-Smad4 (s534708 and s8404), si-TGFβRIII (s24 and s26), si-TAK1 (s13766 and s13767), si-TRAF6 (s14388 and s143789), si-PKCζ (s11128 and s71714), si-PKCι (s11110 and s71706), si-ULK1 (s15963 and s15965) and si-ULK2 (s18704 and s18705). Every experiment was conducted using both siRNAs; the first siRNA listed for each target was used in the main Figures and the second siRNA listed for each target was used in the Supplementary Figures as described.

### Cell Culture and Transfections

A549 cells and H1299 NSCLC cell lines were cultured in a humidified tissue incubator at 37°C under 5% CO_2_. A549 cells and H1299 cells were incubated with Kaighn’s Modification of Hams F-12 (F-12K; Corning, 10-025-CV) and Roswell Park Memorial Institute (RPMI; Corning, 10-043-CVR) media, respectively. Cells were treated with 250 pM TGFβ1, 10 μM ULK-101, 10 μM Compound C, 20 μM SB431542, 40 μM LY294002 and 10 μM P38 MAPK Inhibitor in media supplemented with 10% FBS. Transient siRNA knockdowns were performed using Lipofectamine RNAiMAX (Thermo Fisher Scientific, 13778150) and optimem media (Thermo Fisher Scientific, 22600134) as per the manufacturer’s protocol. Stable GFP-LC3-RFP-LC3ΔG expressing cells were generated using PolyJet transfection reagent (Froggabio, Toronto, ON, Canada) and a cDNA pMRX-IP-GFP-LC3-RFP-LC3ΔG vector (Addgene, 84573). Transfected cells were isolated using growth media supplemented with 10% FBS and 1 μg/mL puromycin (Thermo Fisher Scientific, A1113802).

### Immunoblotting

TNTE lysis buffer (50 mM Tris pH 7.5, 150 mM sodium chloride, 1 mM ethylenediaminetetraacetic acid, 0.5% Triton X-100, 1 mg/mL pepstatin, 50 μM phenylmethylsulfonyl fluoride, 2.5 mM sodium fluoride, and 10 mM sodium pyrophosphate phosphatase inhibitor) was used to lyse cells for 20 min prior to protein collection. Following lysis, cell lysates were centrifuged at 21,000 g_av_ at 4°C for 10 min. Protein concentration was determined using the DC^TM^ protein assay (Bio-Rad, Hercules, CA, United States) and a Victor 3V Multi-Detection Microplate Reader (PerkinElmer, Waltham, MA, United States). Prior to immunoblotting, Laemmli loading buffer was added to the protein lysates and the samples were separated via sodium dodecyl sulfate polyacrylamide gel electrophoresis (SDS-PAGE). Following a standard wet transfer protocol, proteins were transferred onto a nitrocellulose membrane and blocked with 5% skim milk for 1-h, rocking at room temperature. Primary antibodies were incubated overnight with the nitrocellulose membranes, rocking at 4°C. On the following day, nitrocellulose membranes were incubated with the appropriate HRP-conjugated secondary antibody for 1-h at room temperature. Enhanced chemiluminescent substrate (Bio-Rad, 1705060) was added prior to visualizing using a Versa-doc Imager (Bio-Rad) and QuantityOne^®^ 1-D Analysis software (Bio-Rad) analyzed the relative intensity of protein bands.

### Immunofluorescence Microscopy

A549 cells cultured on glass coverslips were treated with 0 or 250 pM TGFβ for 24 h. Following treatment, the cells were washed with PBS, fixed with 4% paraformaldehyde for 10 min, permeabilized after 5 min of 0.1% Triton X-100 and blocked for 1 h. Antibodies against ULK1 were diluted to a final concentration of 1:100. The cells were left in 4°C rocking with the antibody overnight. The following day, the cells were washed with PBS and incubated with an anti-rabbit secondary antibody for 1 h. The cells were washed with PBS and incubated with DAPI dissolved in a PBS solution for 10 min. Coverslips were then mounted onto microscope slides using Immu-mount (Thermo Fisher Scientific, 9990402) and were left in the dark overnight. The coverslips were visualized and imaged using an inverted Olympus IX81 fluorescence microscope or a Nikon Eclipse Ti2 (Nikon Instruments) confocal microscope. ImageJ (version 2.0) was used to quantify relative nuclear ULK1 intensity/Total ULK1 intensity. This experiment was repeated in A549 cells treated with si-RNA against Smad4 and A549 cells treated with si-RNA against TAK1 and TRAF6 in combination with a p38 MAPK inhibitor. Each data point represents quantitation from ≥100 cells from each condition.

### Autophagic Flux Assay

A549 cells and H1299 cells were transfected with a cDNA pMRX-IP-GFP-LC3-RFP-LC3ΔG vector developed by the Mizushima laboratory (30; Addgene). Successfully transfected cells express two forms of LC3: GFP-LC3 and a mutant LC3 with a C-terminal glycine deletion (RFP-LC3ΔG). Immunoblotting using LC3 specific antibodies could distinguish the RFP-LC3ΔG, GFP-LC3-I and GFP-LC3-II bands, which are quantified using QuantityOne^®^ 1-D Analysis software to determine the GFP/RFP ratio. Furthermore, using a 63x objective of an Olympus IX 81 inverted fluorescence microscope, we imaged the Hoechst, green and red channels. The GFP/RFP ratio was determined by ImageJ version 2.0, which quantified the average pixel intensity for green and red channels.

### Assessing Autophagosome and Lysosome Co-localization

A549 cells stably expressing GFP-LC3 were treated with si-RNA against Smad4 or si-RNA targeting TRAF6 and TAK1 in combination with a p38 MAPK inhibitor for 24 h. Each experiment was conducted in the presence and absence of TGFβ1 for 24 h. LysoTracker Deep Red labeled lysosomes and Hoechst stain labeled the nucleus 2 h and 10 min prior to imaging, respectively. Imaging and quantitation was performed as previously described (Trelford and Di Guglielmo, 2020).

### LC3 Puncta

A549 cells expressing GFP-LC3 that were subjected to live imaging using an Olympus IX 81 inverted fluorescence microscope to assess autophagic flux. This also allowed for the determination of the relative LC3 puncta per cell. Image J version 2.0 was used to quantify the number of puncta/cell utilizing puncta size, pixel count and circularity.

### Statistical Analysis

A Student’s *t*-test and One-way or Two-way ANOVA followed by a Dunnett’s multiple comparisons test were used to evaluate the significance of the results. Statistical analyses were performed using GraphPad Prism Software version 9.0 and *P*-values < 0.05 were considered to be statistically significant.

## Results

### TGFβ1 Activates Autophagy by Regulating the mTOR-ULK1 Pathway

**FIGURE 1:**
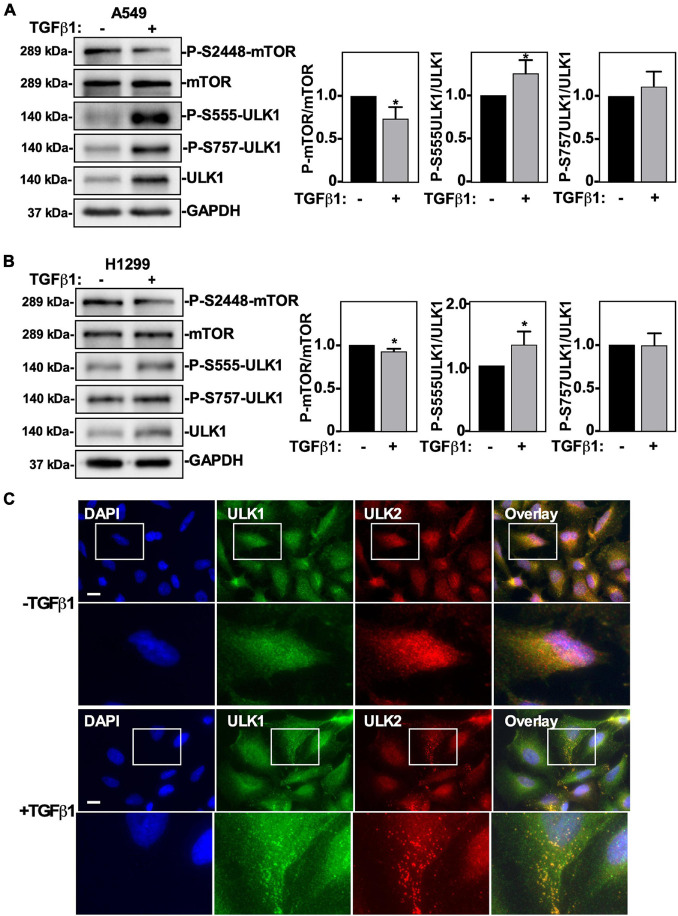
The effect of TGFβ1 on mTOR and ULK1 activity in NSCLC cells. A549 **(A)** or H1299 **(B)** cells were treated with 250 pM TGFβ1 for 24 h. Cells were lysed and subjected to SDS-PAGE and immunoblotting for anti-mTOR, anti-phospho-S2448-mTOR, anti-ULK1, anti-phospho-S555-ULK, anti-phospho-S757-ULK1, or anti-GAPDH (loading control) antibodies. The steady-state levels of phospho-S555-ULK1, ULK1, phospho-S2448-mTOR and mTOR were quantified using QuantityOne software and the phospho-mTOR/mTOR and phospho-ULK1/ULK1 ratios were graphed (*n* = 3 ± SD). Significance is indicated as * = *P* < 0.05. **(C)** A549 cells were treated with 250 pM TGFβ1 for 24 h. Cells were fixed and stained with DAPI (blue), antibodies against ULK1 (green) and ULK2 (red), and imaged using an Olympus IX 81 inverted fluorescence microscope. Bar = 10 μm.

We previously reported that TGFβ1 induced ULK1 protein levels and stimulated autophagy in NSCLC cells (Trelford and Di Guglielmo, 2020), however the mechanism of how this was achieved remained unknown. To this end, we first investigated if TGFβ1 alters AMPK and mTOR activity by following site-specific ULK1 phosphorylation. Briefly, we measured ULK1 S555 phosphorylation to assess AMPK-dependent activity, ULK1 S757 to measure mTOR-dependent phosphorylation of ULK1 and mTOR S2448 phosphorylation to assess active mTOR (Klionsky et al., 2016). A549 cells and H1299 NSCLC cells were treated with TGFβ1 for 24 h prior to lysis and immunoblotting, and we observed that in response to TGFβ1, ULK1 phosphorylation of S555 tripled in A549 cells (Figure 1A) and doubled in H1299 cells (Figure 1B). Although there was a twofold increase in ULK1 protein levels in both cell lines, the ratio of phospho-S555-ULK1/ULK1 rose significantly (Figures 1A,B). Furthermore, we observed that TGFβ1 had little effect on mTOR protein levels but produced a slight, yet significant, decrease in P-mTOR in both A549 and H1299 cells (Figures 1A,B). Since a low P-mTOR/mTOR ratio increases the amount of ULK1 available for AMPK-dependent S555 phosphorylation and a high phospho-S555-ULK1/ULK1 ratio indicates an increase of active ULK1, we postulated that TGFβ1 increases the amount of post-translationally modified ULK1 to initiate autophagy. One hallmark of autophagy is the cellular redistribution of ULK1 to omegasomes (Karanasios et al., 2013). Therefore, to investigate if TGFβ1 alters the subcellular localization of ULK1 and ULK2, we carried out immunofluorescence microscopy (Figure 1C). We observed that TGFβ1 treatment induces a co-localization of both ULK1 and ULK2 in cytoplasmic puncta (Figure 1C). Interestingly, in response to TGFβ1, we also observed a small, but reproducible decrease in the nuclear signal for both ULK1 and ULK2. To confirm this observation, we carried out confocal microscopy and observed an approximate 20% decrease in nuclear ULK1 and ULK2 in response to TGFβ1 (Supplementary Figure 1).

**FIGURE 2:**
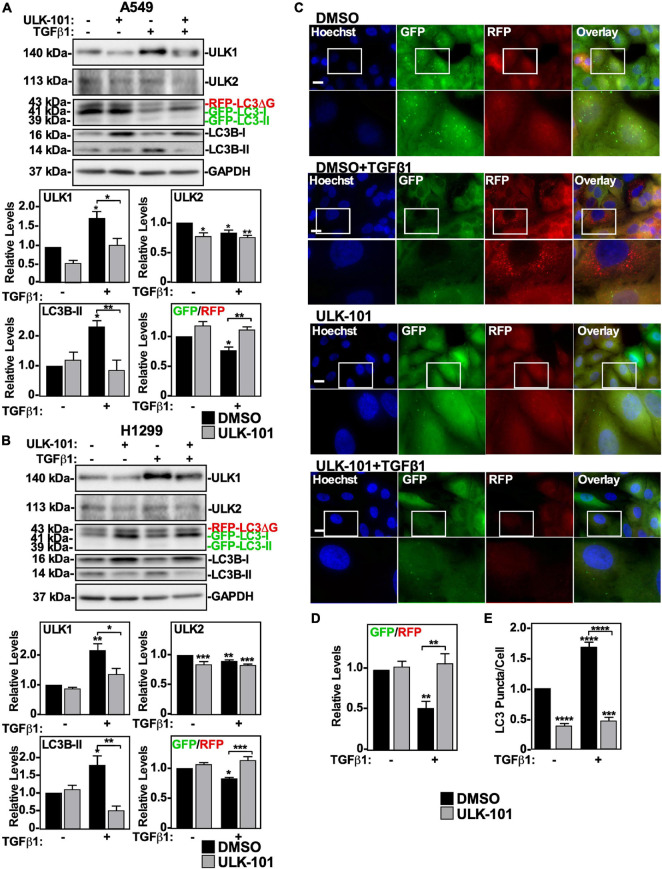
The effect of inhibiting ULK1 on TGFβ1-dependent autophagy in NSCLC cell lines. A549 **(A)** or H1299 **(B)** cells stably expressing GFP-LC3-RFP-LC3ΔG were treated with 10 μM of the ULK1/2 inhibitor, ULK-101, or DMSO (vehicle control) in the presence and absence of 250 pM TGFβ1 for 24 h. Cells were lysed and subjected to SDS-PAGE and immunoblotting for anti-ULK1, anti-ULK2, anti-LC3B and anti-GAPDH antibodies. Quantitative analysis of steady state ULK1, ULK2, and LC3B-II protein levels and the GFP/RFP ratio are shown graphically below representative immunoblots (*n* = 3 ± SD). Significance is indicated as * = *P* < 0.05, ** = *P* < 0.01, and *** = *P* < 0.001. **(C)** A549 cells stably expressing GFP-LC3-RFP-LC3ΔG were treated as described above. Hoechst stain (blue) was added 10 min prior to imaging with a 63x objective using an Olympus IX 81 inverted fluorescence microscope. Bar = 10 μm. **(D)** ImageJ was used to quantify the green and red pixel intensity, and the GFP/RFP ratio is shown graphically below representative images (*n* = 3 ± SD). Significance is indicated as ** = *P* < 0.01. **(E)** Cells and number of puncta/cell were counted using ImageJ version 2.0 software. The data were graphed and shown below representative images (*n* = 3 ± SD). Significance is indicated as *** = *P* < 0.001 and **** = *P* < 0.0001.

To assess the role of ULK1 and/or ULK2 in TGFβ-dependent autophagy, we used ULK-101, a pharmacological inhibitor of both ULK1 and ULK2 (Martin et al., 2018). For this analysis, we utilized A549 cells and H1299 cells stably expressing a GFP-LC3-RFP-LC3ΔG construct that measures autophagic flux, as previously described (Trelford and Di Guglielmo, 2020). These cells were treated with ULK-101 in the presence and absence of TGFβ1, and in both cell lines we observed that TGFβ1 increased ULK1 and LC3B-II protein levels whereas it decreased ULK2 protein levels and the GFP/RFP ratio. Interestingly, ULK-101 decreased ULK1, ULK2 and LC3B-II protein levels and inhibited TGFβ-dependent autophagy, as measured by the GFP/RFP ratio (Figures 2A,B). To further assess autophagic flux in control or ULK-101-treated A549 cells in the presence or absence of TGFβ1 we carried out fluorescence microscopy analysis (Figure 2C). We observed that TGFβ1 significantly decreased the GFP/RFP ratio by 50 ± 10% and that ULK-101 restored the GFP/RFP ratio to control levels (Figure 2D). Furthermore, we quantified LC3-puncta/cell and observed that although TGFβ1 increased the number of LC3-puncta/cell, ULK-101 decreased the ratio of LC3-puncta/cell in the presence and absence of TGFβ1 (Figure 2E).

**FIGURE 3:**
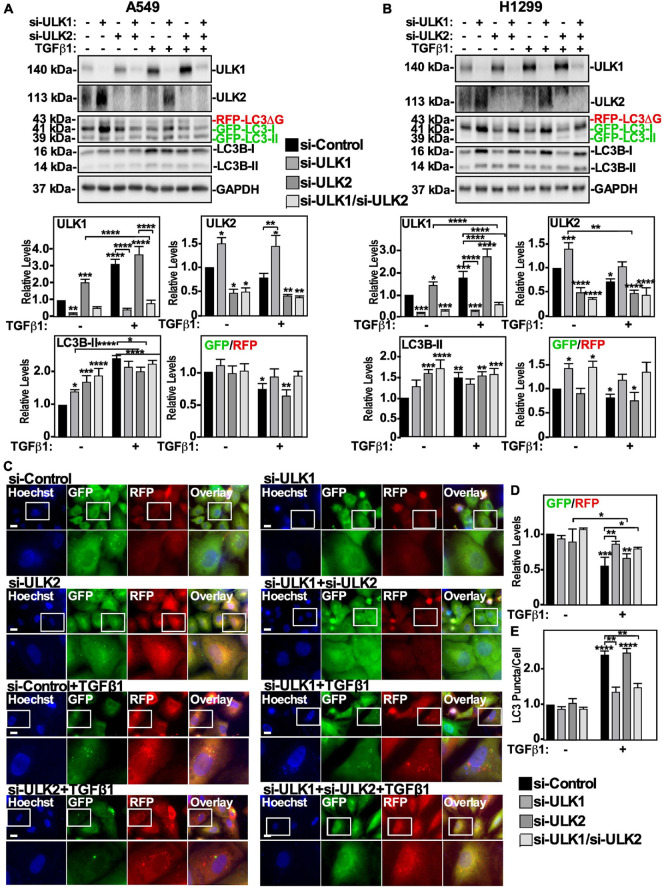
Assessing ULK1 and ULK2 silencing on TGFβ1-dependent autophagy in NSCLC cell lines. A549 **(A)** or H1299 **(B)** cells stably expressing GFP-LC3-RFP-LC3ΔG were transfected with control siRNA (si-Control), siRNA targeting ULK1 (si-ULK1; s15963) or siRNA targeting ULK2 (si-ULK2; s18704) for 48 h. The cells were incubated in the absence or presence of 250 pM TGFβ1 for 24 h, lysed and subjected to SDS-PAGE and immunoblotted for anti-ULK1, anti-ULK2, anti-LC3B and anti-GAPDH antibodies. Quantitative analysis of steady state ULK1, ULK2 and LC3B-II protein levels and the GFP/RFP ratio are shown graphically below representative immunoblots (*n* = 3 ± SD). Significance is indicated as * = *P* < 0.05, ** = *P* < 0.01, *** = *P* < 0.001, and **** = *P* < 0.0001. **(C)** A549 cells stably expressing GFP-LC3-RFP-LC3ΔG were treated as described above. Hoechst stain (blue) was added 10 min prior to imaging with a 63x objective using an Olympus IX 81 inverted fluorescence microscope. Bar = 10 μm. **(D)** ImageJ was used to quantify the green and red pixel intensity, and the GFP/RFP ratio is shown graphically below representative images (*n* = 3 ± SD). Significance is indicated as * = *P* < 0.05, ** = *P* < 0.01, and *** = *P* < 0.001. **(E)** Cells and number of puncta/cell were counted using ImageJ version 2.0 software. The data were graphed and shown graphically below representative images (*n* = 3 ± SD). Significance is indicated as ** = *P* < 0.01 and **** = *P* < 0.0001.

Since ULK-101 inhibits the kinase activity of both ULK1 and ULK2, we next specifically targeted ULK1 or ULK2 using small interfering RNA (siRNA). A549 cells or H1299 cells were treated with control siRNA (si-Control), siRNA targeting ULK-1 (si-ULK1) or ULK-2 (si-ULK2), or a combination of both si-ULK1 and si-ULK2 followed by TGFβ1 stimulation. Western blotting indicated that in A549 cells, two different siRNAs targeting ULK1 significantly decreased ULK1 protein levels by >80% and increased ULK2 and LC3B-II protein levels by 150 ± 18% and 140 ± 9%, respectively (Figure 3A and Supplementary Figure 2A). Furthermore, in the presence of TGFβ1, the two ULK1 siRNAs increased the GFP/RFP ratio compared to the TGFβ1 treatment, suggesting that ULK1 activity is necessary for TGFβ1-induced autophagy in A549 cells (Figure 3A and Supplementary Figure 2A). In H1299 cells, the ULK1 siRNAs also reduced ULK1 protein levels by >80% and increased ULK2 protein levels by 150 ± 21% (Figure 3B and Supplementary Figure 2B). Additionally, the ULK1 siRNAs had no effect on LC3B-II protein levels but consistent with A549 cells, increased the GFP/RFP ratio, suggesting that ULK1 activity is important for TGFβ1-induced autophagy in H1299 cells as well (Figure 3B and Supplementary Figure 2B). In both cell lines, the ULK2 siRNAs decreased ULK2 protein levels, increased LC3B-II and ULK1 protein levels but had no effect on the GFP/RFP ratio (Figures 3A,B and Supplementary Figures 2A,B). Taken together, these results suggest that ULK1 but not ULK2 is involved with TGFβ1-induced autophagy. As a parallel approach, we carried out fluorescence microscopy on GFP-LC3-RFP-LC3ΔG expressing cell lines (Figure 3C). A549 cells transfected with two different siRNA to ULK1 significantly increased the GFP/RFP ratio by 35 ± 9%, whereas si-ULK2 had little effect (Figure 3D and data not shown). Finally, quantifying LC3 puncta/cell revealed that in the presence of TGFβ1, all treatments with siRNAs targeting ULK1 had fewer LC3 puncta/cell (Figure 3E and data not shown). Taken together, our results suggest that TGFβ1 activates autophagy by increasing AMPK-dependent ULK1 S555 phosphorylation.

### TGFβ1-Induced Autophagy Relies on TβRI Kinase Activity

**FIGURE 4:**
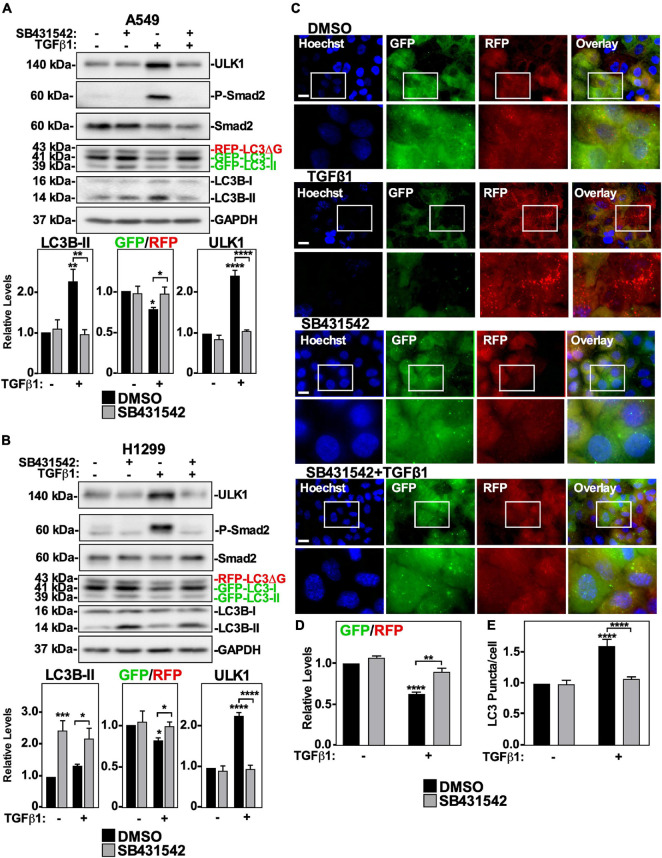
The effect of SB431542 on TGFβ1 induced autophagy in NSCLC cell lines. A549 **(A)** or H1299 **(B)** cells stably expressing GFP-LC3-RFP-LC3ΔG were treated with 20 μM SB431542 or DMSO (vehicle control) in the presence and absence of 250 pM TGFβ1 for 24 h. Cells were lysed and subjected to SDS-PAGE and immunoblotting for anti-ULK1, anti-LC3B and anti-GAPDH antibodies. Quantitative analysis of steady state ULK1 and LC3B-II protein levels and the GFP/RFP ratio are shown graphically below representative immunoblots (*n* = 3 ± SD). Significance is indicated as * = *P* < 0.05, ** = *P* < 0.01, *** = *P* < 0.001, and **** = *P* < 0.0001. **(C)** A549 cells stably expressing GFP-LC3-RFP-LC3ΔG were treated as described above. Hoechst stain (blue) was added 10 min prior to imaging with a 63x objective using an Olympus IX 81 inverted fluorescence microscope. Bar = 10 μm. **(D)** ImageJ was used to quantify the green and red pixel intensity, and the GFP/RFP ratio is shown graphically below representative images (*n* = 3 ± SD). Significance is indicated as ** = *P* < 0.01 and **** = *P* < 0.0001. **(E)** Cells and number of puncta/cell were counted using ImageJ version 2.0 software. The data were graphed and shown below representative images (*n* = 3 ± SD). Significance is indicated as **** = *P* < 0.0001.

Although we determined that TGFβ1-dependent autophagy is facilitated by ULK1, the signaling pathway that effects ULK1 protein levels and phosphorylation are unknown. We therefore assessed which TGFβ receptors are essential to TGFβ1-dependent autophagy. We first inhibited the TβRII/TβRI complex (Miyazawa and Miyazono, 2017) using the a pharmacological inhibitor, SB431542, which blocks the kinase activity of TβRI (Inman et al., 2002). A549 cells and H1299 cells were treated with SB431542 in the presence and absence of TGFβ1 and immunoblotted for ULK1, phospho-Smad2, Smad2 and LC3B. We observed that SB431542 inhibited TGFβ1-dependent Smad2 phosphorylation in both cell lines (Figures 4A,B). In A549 cells, SB431542 blocked the TGFβ1-dependent decrease of the GFP/RFP ratio and increase of ULK1 and LC3B-II protein levels (Figure 4A). In H1299 cells, SB431542 disrupted the TGFβ1-dependent decrease of the GFP/RFP ratio and increase of ULK1 protein levels. However, SB431542 treatments significantly increased LC3B-II protein levels by 210 ± 29% compared to control (Figure 4B). To confirm that TβRI kinase activity is necessary for TGFβ1-induced autophagy, we next utilized fluorescence microscopy to visualize cells expressing GFP-LC3-RFP-LC3ΔG as described above (Figure 4C). Quantifying the GFP and RFP channels revealed that SB431542 increased the TGFβ-dependent GFP/RFP ratio by 30 ± 5%, indicating that SB431542 inhibited TGFβ-dependent autophagic flux (Figure 4D). After analyzing the LC3 puncta/cell using the fluorescence images, we determined that SB431542, in the presence of TGFβ1, decreased the amount of LC3 puncta/cell with respect to the TGFβ1 treatment (Figure 4E). Finally, to assess any involvement of the type III TGFβ receptor (TβRIII), we used an siRNA approach, as this receptor does not have any intrinsic enzymatic activity. Interestingly, A549 cells and H1299 cells expressing two different siRNAs targeting TβRIII exhibited a slightly higher basal level of autophagic flux, but TGFβ-dependent autophagy remained unperturbed by TβRIII silencing (data not shown). Taken together, these results confirm that the activity of the TβRII/TβRI TGFβ receptor complex is necessary for the TGFβ1-dependent increase of autophagic flux in both NSCLC cell lines.

### Smad4-Dependent TGFβ1 Signaling Activates Autophagy

**FIGURE 5:**
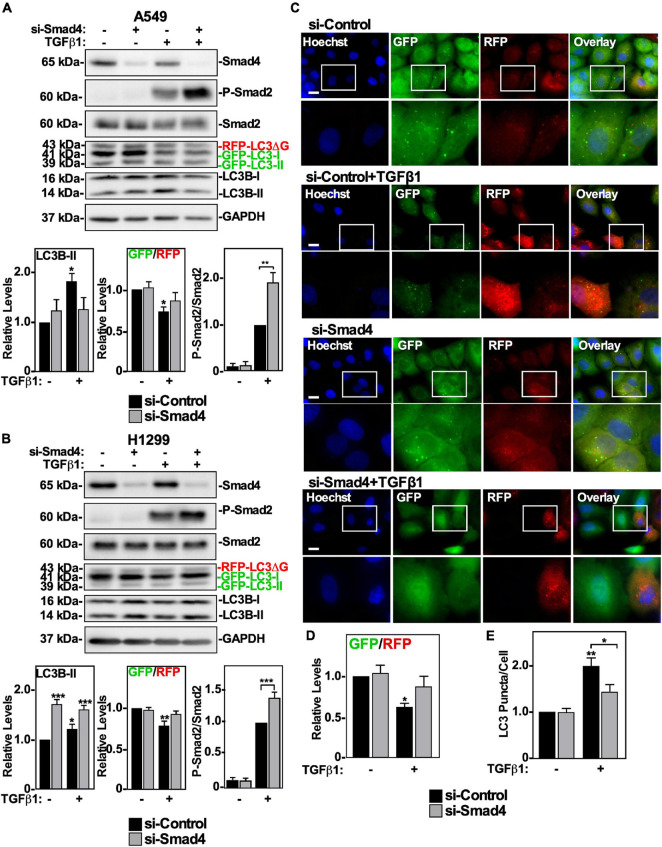
The effect of Smad4 silencing on TGFβ1-dependent autophagy in NSCLC cell lines. A549 **(A)** or H1299 **(B)** cells stably expressing GFP-LC3-RFP-LC3ΔG were transfected with si-Control or siRNA targeting Smad4 (si-Smad4; s534708) for 48 h. The cells were incubated in the absence or presence of 250 pM TGFβ1 for 24 h, lysed and subjected to SDS-PAGE and immunoblotted for anti-Smad4, anti-P-Smad2, anti-Smad2, anti-LC3B and anti-GAPDH antibodies. Quantitative analysis of steady state LC3B-II protein levels and the GFP/RFP ratio are shown graphically below representative immunoblots (*n* = 3 ± SD). Significance is indicated as * = *P* < 0.05, ** = *P* < 0.01, and *** = *P* < 0.001. **(C)** A549 cells stably expressing GFP-LC3-RFP-LC3ΔG were treated as described above. Hoechst stain (blue) was added 10 min prior to imaging with a 63x objective using an Olympus IX 81 inverted fluorescence microscope. Bar = 10 μm. **(D)** ImageJ was used to quantify the green and red pixel intensities, and the GFP/RFP ratio is shown below representative images (*n* = 3 ± SD). Significance is indicated as * = *P* < 0.05. **(E)** Cells and number of puncta/cell were counted using ImageJ version 2.0 software. The data were graphed and shown below representative images (*n* = 3 ± SD). Significance is indicated as * = *P* < 0.05 and ** = *P* < 0.01.

After TGFβ1 binds to TβRII/TβRI complexes, it initiates canonical and non-canonical signaling (Gunaratne et al., 2012). Since we observed that inhibiting TGFβ receptor kinase activity and Smad2 phosphorylation resulted in inhibition of autophagy (Figure 4), we assessed if reducing the accessibility of Smad2 to the TGFβ receptor complex would affect TGFβ-dependent autophagy. This was carried out by siRNA-mediated silencing of the Smad Anchor necessary for Receptor Activation (SARA). Interestingly, reducing SARA levels in both A549 or H1299 cells did not inhibit TGFβ-dependent induction of LC3B-II protein levels, or inhibit autophagy (data not shown). These results suggest that if the canonical TGFβ signaling pathway results in autophagy, removing a major member of the pathway, such as Smad4, may be necessary to alter TGFβ-dependent autophagy. We therefore evaluated if Smad4 silencing via siRNA targeting (si-Smad4) influenced TGFβ1-dependent autophagy. A549 cells and H1299 cells were transfected with si-Control or two different siRNAs targeting Smad4 in the presence or absence of TGFβ1, lysed and immunoblotted for Smad4, P-Smad2, Smad2, and LC3B. In both cell lines, we observed that TGFβ1 increased LC3B-II protein levels and decreased the GFP/RFP ratio (Figures 5A,B and Supplementary Figures 3A,B). Interestingly, Smad4 silencing increased the proportion of phosphorylated Smad2, which suggested that TGFβ-dependent autophagy relies on the presence of Smad4 (Figures 5A,B). Indeed, although Smad4 silencing had differing effects on LC3B-II protein levels in A549 *vs.* H1299 cells, both cell lines showed attenuated TGFβ1-dependent GFP/RFP ratio in the absence of Smad4, suggesting that Smad4 is necessary to induce TGFβ-dependent autophagic flux (Figures 5A,B and Supplementary Figures 3A,B). To investigate this further, we used fluorescence microscopy to image the GFP/RFP autophagic flux ratio in A549 cells (Figure 5C). Quantifying the GFP/RFP ratios indicated that TGFβ1 decreased the GFP/RFP ratio compared to the si-Control treatment by 60 ± 5%. Alternatively, siRNAs targeting Smad4 in the presence of TGFβ1 did not significantly alter the GFP/RFP ratio with respect to the si-Control treatment (Figure 5D and data not shown). Lastly, we examined the influence that Smad4 had on relative LC3 puncta/cell. Although the TGFβ1 treatment significantly increased the relative number of LC3 puncta/cell, we observed that TGFβ1 treatment in Smad4-silenced cells did not significantly increase the ratio of LC3 puncta/cell compared to control cells (Figure 5E and data not shown). These results support the conclusion that TGFβ1 induces autophagy via Smad4. Having ascertained that the canonical pathway is important for promoting TGFβ1-dependent autophagy, we next assessed the contribution of non-canonical TGFβ pathways.

### Non-canonical TGFβ1 Signaling Upregulates Autophagy

**FIGURE 6:**
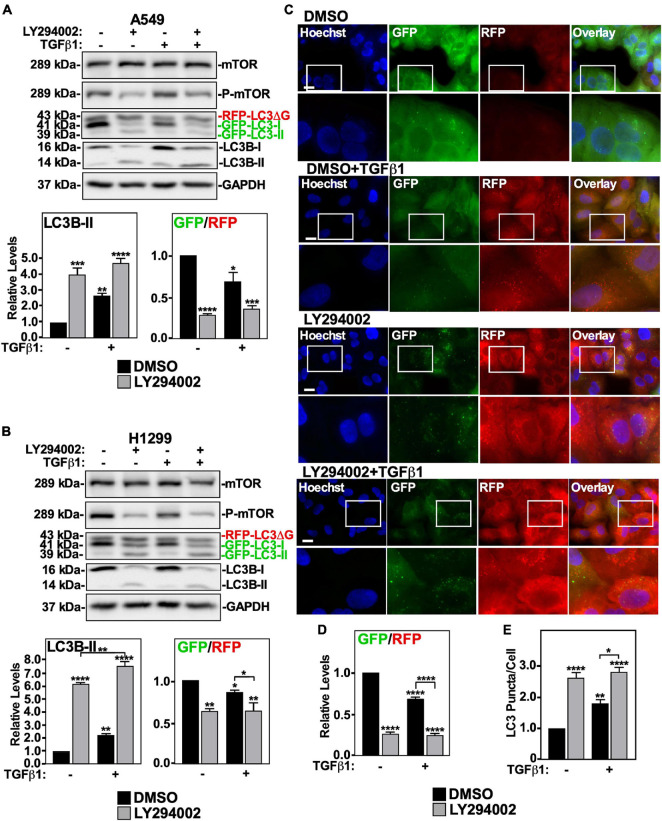
The effect of LY294002 on TGFβ1 induced autophagy in NSCLC cell lines. A549 **(A)** or H1299 **(B)** cells stably expressing GFP-LC3-RFP-LC3ΔG were treated with 40 μM LY294002 or DMSO (vehicle control) in the presence and absence of 250 pM TGFβ1 for 24 h. Cells were lysed and subjected to SDS-PAGE and immunoblotting for anti-ULK1, anti-LC3B and anti-GAPDH antibodies. Quantitative analysis of steady state ULK1 and LC3B-II protein levels and the GFP/RFP ratio are shown below representative immunoblots (*n* = 3 ± SD). Significance is indicated as * = *P* < 0.05, ** = *P* < 0.01, *** = *P* < 0.001, and **** = *P* < 0.0001. **(C)** A549 cells stably expressing a cDNA GFP-LC3-RFP-LC3ΔG construct were treated as described above. Hoechst stain (blue) was added 10 min prior to imaging with a 63x objective using an Olympus IX 81 inverted fluorescence microscope. Bar = 10 μm. **(D)** ImageJ was used to quantify the green and red pixel intensities, and the GFP/RFP ratio is shown below representative images (*n* = 3 ± SD). Significance is indicated as **** = *P* < 0.0001. **(E)** Cells and number of puncta/cell were counted using ImageJ version 2.0 software. The data were graphed and shown below representative images (*n* = 3 ± SD). Significance is indicated as * = *P* < 0.05, ** = *P* < 0.01, and **** = *P* < 0.0001.

We first investigated the role of the PI3K non-canonical TGFβ signaling pathway on TGFβ1-induced autophagy using LY294002, an inhibitor of the PI3K-mTOR pathway (Zhang et al., 2005; Ding et al., 2010). A549 cells and H1299 cells were treated with LY294002 in the presence and absence of TGFβ1, lysed and immunoblotted for mTOR, P-mTOR, and LC3B. We observed that LY294002 treatment increased LC3B-II protein levels and reduced the GFP/RFP ratio to a greater extent than the TGFβ1 treatment alone (Figures 6A,B). These results suggest that the PI3K pathway and autophagic flux are inversely proportional to one another. We verified that the PI3K pathway does not facilitate TGFβ1-dependent autophagy by treating A549 cells with LY294002, with and without TGFβ1, prior to fluorescence microscopy imaging (Figure 6C). In all cases where the cells were treated with LY294002, we observed a marked decrease in GFP-LC3 signal, and the quantitation of the GFP/RFP ratios suggested that LY294002 decreased the GFP/RFP ratio in the presence and absence of TGFβ1 (Figure 6D). Finally, we observed that both TGFβ1 and LY294002 increased the amount of LC3 puncta/cell, however LY294002 significantly increased (>50%) the number of LC3 puncta/cell compared to the TGFβ1 treatment alone (Figure 6E). Since these results suggest that any PI3K activity that is stimulated by TGFβ would impede autophagy, we next turned our attention to another non-canonical TGFβ pathway, the aPKC pathway.

Both aPKCζ and aPKCι have been shown to be involved with TGFβ-dependent processes such as EMT and apoptosis (Gunaratne and Di Guglielmo, 2013; Gunaratne et al., 2014). To investigate if this pathway is involved with autophagy, we utilized siRNAs selective for aPKCζ (si-aPKCζ) or aPKCι (si-aPKCι). Since we had previously observed that aPKCι silencing increases aPKCζ protein levels (Gunaratne et al., 2014), we utilized a double si-aPKCι and si-aPKCζ knockdown approach. A549 cells and H1299 cells were treated with si-Control or si-aPKCζ/si-aPKCι in the presence or absence of TGFβ1, lysed and immunoblotted for aPKCζ, aPKCι, LC3B, and GAPDH. In A549 cells, we observed that TGFβ1 increased LC3B-II protein levels and decreased the GFP/RFP ratio in the presence of si-Control and si-aPKCζ/si-aPKCι treatments (Supplementary Figure 4A). In H1299 cells, we found that si-aPKCζ/si-aPKCι and TGFβ1 increased LC3B-II protein levels compared to the si-Control treatment, however, in the presence of TGFβ1, si-aPKCζ/si-aPKCι significantly reduced LC3B-II protein levels by 25 ± 5%. Additionally, in the presence of TGFβ1, the GFP/RFP ratio of the si-aPKCζ/si-aPKCι treatment was not statistically different compared to the si-Control treatment (Supplementary Figure 4B). Based on these results, aPKCs may not be involved with TGFβ1-dependent autophagy. To confirm this, we treated A549 cells with si-Control or si-aPKCζ/si-aPKCι and used fluorescence microscopy to image GFP-LC3 and RFP-LC3ΔG (Supplementary Figure 4C). Quantitation of the GFP/RFP ratios indicated that all TGFβ1 treatments had reduced GFP/RFP ratios with respect to the si-Control treatment (Supplementary Figure 4D), and that TGFβ1 increased the number of LC3 puncta/cell regardless of aPKC knockdown (Supplementary Figure 4E). Having observed that TGFβ1 may not require aPKCζ or aPKCι to activate autophagy, we next directed our attention to the TAK1-tumor necrosis factor receptor-associated factor 6 -P38 mitogen activated protein kinase (TAK1-TRAF6-P38 MAPK) pathway.

**FIGURE 7:**
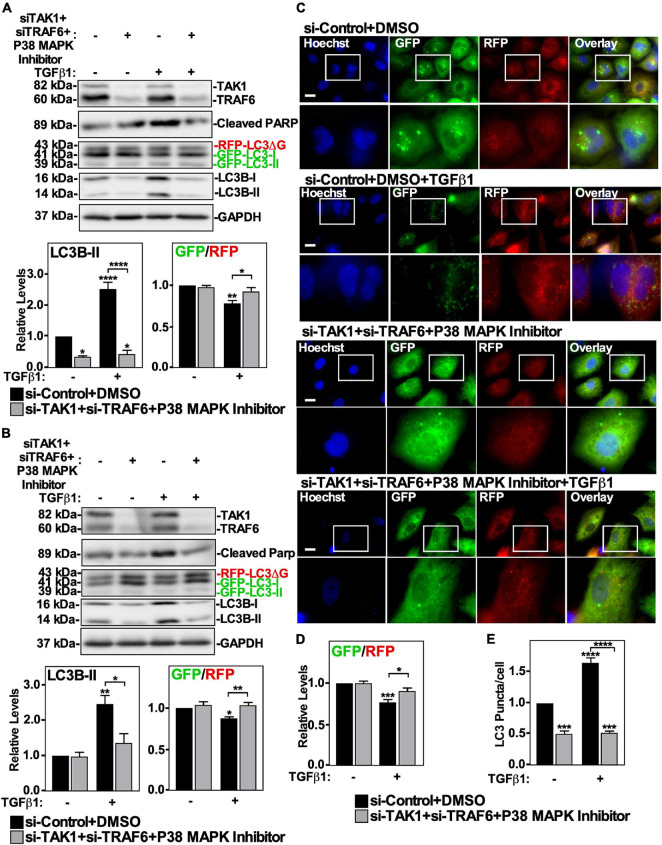
The effect of TAK1-TRAF6-P38 MAPK pathway on TGFβ1-dependent autophagy in NSCLC cell lines. A549 **(A)** or H1299 **(B)** cells stably expressing GFP-LC3-RFP-LC3ΔG were transfected with si-Control or siRNA targeting TAK1 (si-TAK1; s13766), siRNA targeting TRAF6 (si-TRAF6; s14388) for 48 h. The cells were incubated in the absence or presence of 250 pM TGFβ1 and 10 μM P38 MAPK inhibitor for 24 h. The cells were then lysed, subjected to SDS-PAGE and immunoblotted for anti-TAK1, anti-TRAF6, anti-cleaved PARP, anti-LC3B and anti-GAPDH antibodies. Quantitative analysis of steady state LC3B-II protein levels and the GFP/RFP ratio are shown below representative immunoblots (*n* = 3 ± SD). Significance is indicated as * = *P* < 0.05, ** = *P* < 0.01, and **** = *P* < 0.0001. **(C)** A549 cells stably expressing GFP-LC3-RFP-LC3ΔG were treated as described above. Hoechst stain (blue) was added 10 min prior to imaging with a 63x objective using an Olympus IX 81 inverted fluorescence microscope. Bar = 10 μm. **(D)** ImageJ was used to quantify the green and red pixel intensities, and the GFP/RFP ratio is shown below representative images (*n* = 3 ± SD). Significance is indicated as * = *P* < 0.05 and *** = *P* < 0.001. **(E)** Cells and number of puncta/cell were counted using ImageJ version 2.0 software. The data were graphed and shown below representative images (*n* = 3 ± SD). Significance is indicated as *** = *P* < 0.001 and **** = *P* < 0.0001.

To assess if the TAK1-TRAF6-P38 MAPK pathway was involved with TGFβ1-dependent autophagy, we first inhibited each component of the pathway separately. The effects of pharmacologically inhibiting p38 MAPK in A549 cells and H1299 cells was assessed by immunoblotting for cleaved PARP, as TGFβ1 increases PARP cleavage via P38 MAPK (Gunaratne et al., 2015). In both cell lines, we observed that the P38 MAPK inhibitor blocked TGFβ1-dependent PARP cleavage, however it did not alter TGFβ-dependent autophagy, as assessed by western blotting and fluorescence microscopy (Supplementary Figure 5). We next assessed the involvement of TRAF6 in TGFβ-dependent autophagy using siRNA specific for TRAF6 (si-TRAF6). A549 cells and H1299 cells treated with si-Control or si-TRAF6 in the presence or absence of TGFβ1 showed that TRAF6 silencing did not affect TGFβ1 mediated changes to LC3B-II protein levels or the GFP/RFP ratio (Supplementary Figures 6A,B). To verify that TRAF6 silencing had no effect on TGFβ1-induced autophagy, we used fluorescence microscopy on A549 cells treated as described above (Supplementary Figure 6C). In the presence and absence of TGFβ1, si-TRAF6 did not alter the GFP/RFP ratios or impact the number of LC3 puncta/cell (Supplementary Figures 6D,E). Finally, we used siRNA specific for TAK1 (si-TAK1) to silence TAK1 in A549 cells and H1299 cells. In both cell lines, si-TAK1 decreased LC3B-II protein levels and the partially reversed TGFβ-dependent autophagic flux, as assessed by western blotting (Supplementary Figures 7A,B). Although this observation was not seen by fluorescence microscopy (Supplementary Figures 7C–E), the promising results from the western blot analysis prompted us to try a combination of inhibitors of this pathway. We therefore inhibited TAK1, TRAF6, and P38 MAPK activity simultaneously to achieve maximal blockade of this non-canonical TGFβ signaling pathway (Figure 7). A549 cells and H1299 cells were treated with si-TRAF6, si-TAK1 and P38 MAPK inhibitor in the presence and absence of TGFβ1, lysed and immunoblotted for TAK1, TRAF6, cleaved PARP, and LC3B. In both cell lines, we observed that using two sets of siRNAs to TAK1 and TRAF6, in combination with a P38 MAPK inhibitor decreased LC3B-II protein levels and increased the GFP/RFP ratio (Figures 7A,B and Supplementary Figure 8C). To verify the role of this pathway in TGFβ1-induced autophagy, we used fluorescence microscopy to image A549 cells treated as described above (Figure 7C). Quantitation revealed that inhibiting the TAK1-TRAF6-P38 MAPK pathway, in the presence of TGFβ1, significantly increased the GFP/RFP ratio by 20 ± 5% compared to the TGFβ1 treatment (Figure 7D and data not shown). Additionally, in the presence and absence of TGFβ1, inhibiting the TAK1-TRAF6-P38 MAPK pathway reduced the relative number of LC3 puncta/cell (Figure 7E). Taken together, these results suggest that TGFβ1 relies on the TAK1-TRAF6-P38 MAPK to upregulate autophagy.

### TGFβ1-Induced Autophagosome-Lysosome Co-localization Is Regulated by Smad4 and TAK1-TRAF6-P38 MAPK Signaling Pathways

**FIGURE 8:**
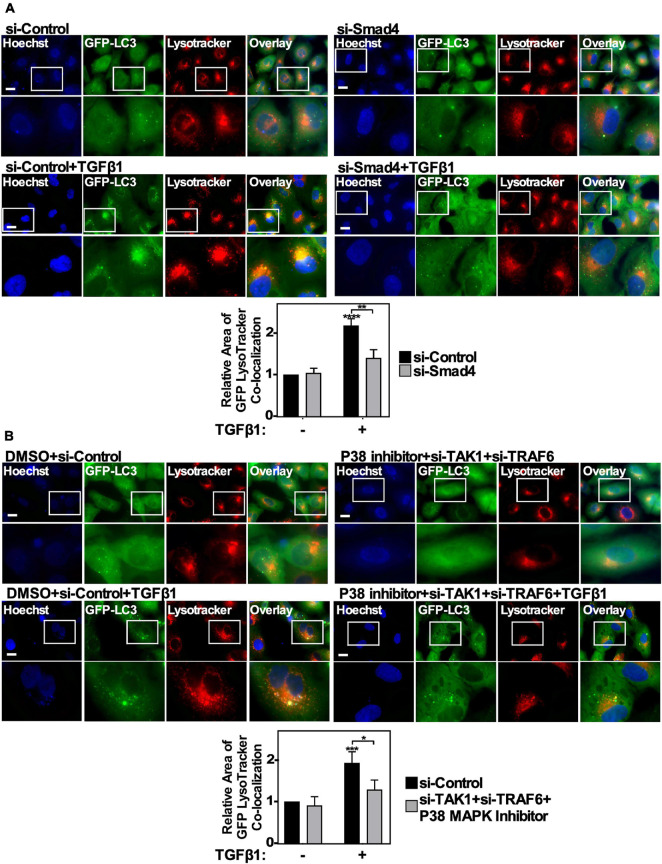
The effect of canonical and non-canonical TGFβ signaling on autophagosome/lysosome co-localization. **(A**) A549 cells expressing GFP-LC3-RFP-LC3ΔG were transfected with si-Control or si-Smad4 (s534708) for 48 h. The cells were then incubated in the absence or presence of 250 pM TGFβ1 for 24 h. LysoTracker Deep Red (red) and Hoechst stain (blue) were added 2 h and 10 min, respectively, prior to imaging. Images were obtained with a 63x objective using an Olympus IX 81 inverted fluorescence microscope. Scale bars = 10 μm. Image J version 2.0 was used to quantify the number of yellow pixels per cell area for each treatment. The data were graphed from 3 independent experiments (mean ± SD). Significance is indicated as ** = *P* < 0.01 and **** = *P* < 0.0001. Bar = 10 μm. **(B)** A549 cells expressing GFP-LC3-RFP-LC3ΔG were transfected with si-Control or si-TAK1 (s13766) and si-TRAF6 (s14388) for 48 h. The cells were then incubated in the absence or presence of 250 pM TGFβ1 and 10 μM P38 MAPK inhibitor for 24 h. LysoTracker Deep Red (red) and Hoechst stain (blue) were added 2 h and 10 min, respectively, prior to imaging. Images were obtained with a 63x objective using an Olympus IX 81 inverted fluorescence microscope. Scale bars = 10 μm. Image J version 2.0 was used to quantify the number of yellow pixels per cell area for each treatment. The data were graphed from 3 independent experiments (mean ± SD). Significance is indicated as * = *P* < 0.05 and *** = *P* < 0.001. Bar = 10 μm.

Above we observed that the Smad4 and TAK1-TRAF6-P38 branches of the canonical and non-canonical TGFβ signaling pathways influence TGFβ-dependent autophagy. To gain more mechanistic insight, we next utilized A549 cells stably expressing GFP-labeled LC3 protein to determine if either Smad4 silencing or inhibiting the TAK1-TRAF6-P38 MAPK pathway disrupted the TGFβ1-dependent increase of GFP-LC3-lysosome co-localization. Briefly, A549 cells expressing GFP-LC3 were transfected with si-Control or si-Smad4, in the presence and absence of 250 pM TGFβ1 for 24 h and labeled with LysoTracker Deep Red to identify lysosomes (Figure 8A). We observed that in the absence of TGFβ there was little GFP-LC3 co-localizing with lysosomes, however TGFβ induced the accumulation of GFP-LC3 into Lysotracker-positive puncta. Interestingly, Smad4 silencing reduced both GFP-LC3 accumulation within cells and the co-localization with lysotracker puncta (Figure 8A). Inhibiting the TAK1-TRAF6-P38 pathway using a combination of si-TAK1, si-TRAF6 and P38 MAPK inhibitor yielded similar results, as inhibiting the TAK1-TRAF6-P38 pathway blocked the TGFβ1-dependent increase in GFP-LC3-lysosome co-localization (Figure 8B). In summary, these results verified that both Smad4 and the TAK1-TRAF6-P38 MAPK signaling pathways are necessary for TGFβ1 to induce autophagosome and lysosome co-localization, which temporally occurs immediately prior to lysosomal-dependent degradation.

### Smad4 Regulates ULK1 Phosphorylation and TAK1-TRAF6-P38 MAPK Activation Inhibits the mTOR-ULK1 Pathway

**FIGURE 9:**
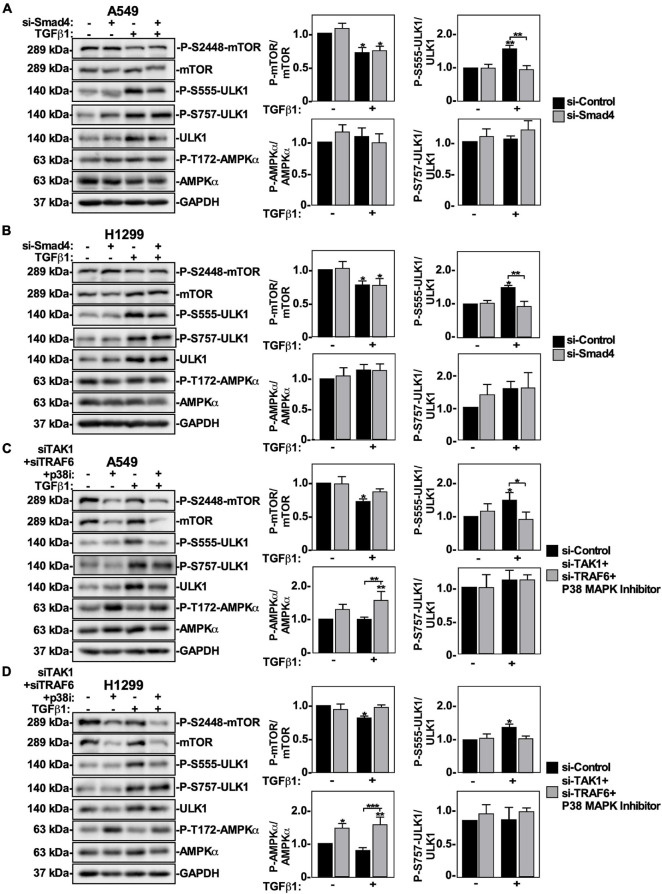
The effect of canonical and non-canonical TGFβ signaling on mTOR and ULK1 activity in NSCLC cells. A549 **(A)** or H1299 **(B)** cells were transfected with si-Control or si-Smad4 (s534708) for 48 h. The cells were incubated in the absence or presence of 250 pM TGFβ1 for 24 h, lysed and subjected to SDS-PAGE and immunoblotted for anti-mTOR, anti-phospho-S2448-mTOR, anti-phospho-S757-ULK1, anti-ULK1, anti-phospho-S555-ULK1, anti-AMPKα, anti-phospho-T172-AMPKα and anti-GAPDH antibodies. The steady-state levels of phospho-S555-ULK1, ULK1, anti-phospho-S757-ULK1, phospho-T172-AMPKα, AMPKα, phospho-S2448-mTOR, and mTOR were quantified using QuantityOne software and the phospho-mTOR/mTOR, phospho-ULK1/ULK1 and phospho-AMPKα/AMPKa ratios were graphed (*n* = 3 ± SD). Significance is indicated as * = *P* < 0.05 and ** = *P* < 0.01. A549 **(C)** or H1299 **(D)** cells were transfected with si-Control or si-TAK1 (s13766) and si-TRAF6 (s14388) for 48 h. The cells were incubated in the absence or presence of 250 pM TGFβ1 and 10 μM P38 MAPK inhibitor (p38i) for 24 h. The cells were then lysed, subjected to SDS-PAGE and immunoblotted for anti-mTOR, anti-phospho-S2448-mTOR, anti-ULK1, anti-phospho-S757-ULK1, anti-phospho-S555-ULK1, anti-AMPKα, anti-phospho-T172-AMPKα, and anti-GAPDH antibodies. The steady-state levels of phospho-S555-ULK1, ULK1, phospho-T172-AMPKα, AMPKα, phospho-S2448-mTOR and mTOR were quantified using QuantityOne software and the phospho-mTOR/mTOR, phospho-AMPKα/AMPKα and phospho-ULK1/ULK1 ratios were graphed (*n* = 3 ± SD). Significance is indicated as * = *P* < 0.05, ** = *P* < 0.01, and *** = *P* < 0.001.

Since TGFβ1 activates autophagy using Smad4 and TAK1-TRAF6-P38 MAPK signaling pathways, we next investigated if these pathways influenced mTOR and ULK1 phosphorylation. A549 cells and H1299 cells were treated with si-Control or two siRNAs targeting Smad4 in the presence or absence of TGFβ1, lysed and immunoblotted using phospho-specific antibodies for mTOR and ULK1. In both cell lines, Smad4 knockdown had no effect on the P-mTOR/mTOR or phospho-S757-ULK1/ULK1 ratios (Figures 9A,B). However, in the presence of TGFβ1, Smad4 silencing decreased the phospho-S555-ULK1/ULK1 in A549 cells by 50 ± 15% and in H1299 cells by 50 ± 19% (Figures 9A,B). To assess if this could be due to increased AMPKα activity, we analyzed AMPKα T172 phosphorylation status and observed that P-AMPKα levels remained constant in the presence or absence of TGFβ and/or Smad4 (Figures 9A,B).

Next, A549 cells and H1299 cells were treated with si-TRAF6, si-TAK1 and P38 MAPK inhibitor in the presence of TGFβ1, lysed and immunoblotted for P-mTOR, mTOR, phospho-S555-ULK1, phospho-S757-ULK1, ULK1 and GAPDH. In both cell lines, inhibiting the TAK1-TRAF6-P38 MAPK pathway had no effect on the phospho-S757-ULK1/ULK1 ratios (Figures 9C,D). In A549 cells treated with TGFβ1, inhibiting the TAK1-TRAF6-P38 MAPK pathway increased the P-mTOR/mTOR ratio by 25 ± 12% and decreased the phospho-S555-ULK1/ULK1 ratio by 20 ± 5% (Figure 9C). In H1299 cells treated with TGFβ1, inhibiting the TAK1-TRAF6-P38 MAPK pathway increased the P-mTOR/mTOR ratio by 20 ± 5% and decreased the phospho-S555-ULK1/ULK1 ratio by 30 ± 10% (Figure 9D). Interestingly, inhibiting the TAK1-TRAF6-P38 pathway increased the basal level of AMPKα-T172 phosphorylation, however the P-AMPKα/AMPKα ratio was unchanged in response to TGFβ (Figures 9C,D). Since these results suggested that AMPKα activity may not be necessary for TGFβ1-dependent autophagy, we inhibited AMPKα activity in A549 cells using Compound C and observed that while Compound C altered basal autophagy, it did not affect TGFβ1-dependent autophagy (Supplementary Figure 9). Finally, to assess if canonical and/or non-canonical pathways would induce the nuclear export of ULK1 in response to TGFβ, we carried out confocal microscopy in cells treated with siRNAs targeting Smad4 or TAK1 + TRAF6, in combination with a P38 inhibitor. We observed that perturbing either pathway inhibits TGFβ1-dependent ULK1 cellular re-localization from the nucleus (Supplementary Figure 10).

**FIGURE 10:**
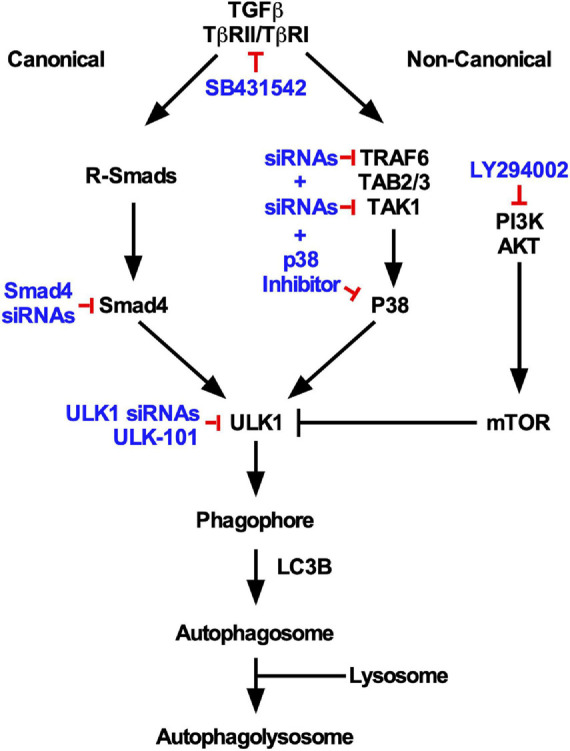
Summary of TGFβ1-dependent autophagy in NSCLC cells. Based on the inhibitory strategies used in this study (indicated in blue), specific canonical and non-canonical TGFβ pathways were observed to regulate TGFβ-dependent autophagy. Both pathways were observed to converge on ULK1 activity and were necessary for lysosomal targeting of LC3B.

Taken together, our results show that by using various pharmacological and/or siRNA mediated approaches, we have observed that TGFβ1 induces autophagy by increasing ULK1 activity, which is dependent on TβRI kinase activity, the canonical Smad4 signaling pathway and the non-canonical TAK1-TRAF6-P38 MAPK signaling pathway (Figure 10).

## Discussion

We previously uncovered several aspects of TGFβ1-dependent autophagy in NSCLC cells, and observed that TGFβ1 increased *ULK1*, *ATG9A*, *ATG16L1* and *LC3* gene expression, but only the protein levels of LC3B-II and ULK1 (Trelford and Di Guglielmo, 2020). We also observed that LC3B-II protein levels are limited in measuring autophagy and therefore methods that investigate autophagic flux should be used to measure TGFβ1-dependent autophagy. Finally, we reported that siRNA-mediated ATG5/7 knockdown decreases TGFβ-dependent autophagic flux in NSCLC cells (Trelford and Di Guglielmo, 2020). Therefore, although macroautophagy can be activated independently of ATG5/7 or ULK1 activity (Arakawa et al., 2017), our results suggest that the majority of autophagic degradation initiated by TGFβ1 is mediated by canonical macroautophagy (Trelford and Di Guglielmo, 2020).

Here, using pharmacological inhibitors and siRNA to target specific TGFβ1 signaling pathways, we mechanistically characterized TGFβ1-dependent autophagy in two NSCLC cell lines. We observed that TGFβ1-dependent autophagy was diminished in the absence of Smad4 protein or the disruption of the TAK1-TRAF6-P38 MAPK pathway. Further analysis revealed that Smad4 knockdown did not alter P-mTOR/mTOR ratios, suggesting that it affects autophagy downstream of mTOR. Consistent with this hypothesis, we found that Smad4 upregulated AMPK-dependent ULK1 S555 phosphorylation. However, due to the fact that Smad4 knockdown did not disrupt the increase of ULK1 protein levels, TGFβ may alter *ULK1* expression or degradation via a Smad4-independent mechanism. Alternatively, the TAK1-TRAF6-P38 MAPK pathway may influence autophagy by impeding mTOR S2448 phosphorylation. This would explain why inhibiting the TAK1-TRAF6-P38 MAPK pathway increased the P-mTOR/mTOR ratio, decreased autophagic flux and reduced the phospho-S555-ULK1/ULK1 ratio.

The link between Smad4 and TGFβ1-dependent autophagy that we observed in NSCLC cells was consistent with studies investigating TGFβ-dependent autophagy in pancreatic ductal adenocarcinoma cell lines (Liang et al., 2020) and breast cancer cell lines (Cheng et al., 2018). However, the role of Smad4 in TGFβ-dependent autophagy is complex and remains an area that needs to be further investigated. This is because Smad4 was observed to not be essential for TGFβ-dependent autophagy in Smad4 negative cell lines (Liang et al., 2020). Also, the presence of Smad4 may not be sufficient to drive TGFβ-dependent autophagy. For example, when we inhibited the TAK1-TRAF6-P38 MAPK pathway, Smad4 did not sustain TGFβ1-dependent autophagy in NSCLC cell lines. Finally, there is some evidence to suggest that Smad4 impedes autophagy. For instance, in orthotopic pancreatic tissue samples, *Smad4* expression was inversely correlated to autophagy (Wang et al., 2018). Therefore, the role of Smad4 in TGFβ-dependent autophagy is likely cell type dependent. In support of this, miRNA targeting of Smad4 in breast cancer cells attenuated autophagy (Cheng et al., 2018) whereas Smad4 depletion protected pancreatic cancer cells from radiotherapy by inducing autophagy (Wang et al., 2018). Less unclear is the importance of Smad4 in tumorigenesis. To date, Smad4 is known as the most common Smad family gene mutated in cancer (Sarshekeh et al., 2017). Smad4 mutations are found in approximately 50% of pancreatic adenocarcinomas (Howe et al., 1998), 20% of colorectal cancers (Chu et al., 2004) and 5% of head and neck squamous cell carcinomas (Lin et al., 2019). Currently, more research is needed to characterize the relationship between Smad4, autophagy and cancer to determine if Smad4 genetic targeting in cancer cells could impede tumorigenesis by hindering both TGFβ and autophagy-dependent drivers of cancer.

Since members of the TAK1-TRAF6-P38 MAPK pathway have been shown to affect autophagy, we decided to study this pathway in TGFβ1-dependent autophagy. A possible explanation for the lack of knowledge with respect to how it is involved in TGFβ1-dependent autophagy, is that this pathway is accessed by numerous stimuli. For instance, TAK1 is activated by tumor necrosis factors, toll-like receptors, interleukins and TGFβ ligands prior to activating P38 MAPK and c-Jun N-terminal kinase, which regulate metabolism, growth, survival and tumorigenesis (Landström, 2010). Polyubiquitination and activation of TRAF6 is initiated by interleukins and toll-like receptors during innate proinflammatory responses; nucleotide-binding and oligomerization domain containing protein 2 receptors recognizing bacteria; recognition of viral RNAs; TGFβ receptors; receptor activator of nuclear factor kappa-B ligands during osteoclast differentiation; and several cell surface receptors on B-lymphocytes and T-lymphocytes (Dainichi et al., 1107). Therefore, due to the broad spectrum of stimuli that induce TAK1, TRAF6, or P38 MAPK activation, we knew little about their respective roles in autophagy and even less with regards to TGFβ1-dependent autophagy.

TAK1 functions as an upstream AMPK kinase by phosphorylating AMPK at threonine172 (Aashaq et al., 2019). For this reason, the increase in P-AMPKα/AMPKα ratio in cells subject to TAK1-TRAF6-P38 MAPK pathway inhibition was surprising. Since AMPK stimulates autophagy by phosphorylating ULK1 to form the ULK1 complex and by suppressing mTOR activity (Liu et al., 2018), TAK1 has become a target of interest to suppress autophagy. For instance, TAK1 inactivation in mice has resulted in the accumulation of dysfunctional mitochondria in skeletal muscle (Hindi et al., 2018). Furthermore, compared to their wild-type counterparts, mice with hepatocyte depletion of *TAK1* developed hepatosteatosis due to autophagy suppression in which further analysis indicated that TAK1 depletion suppressed AMPK activity and increased mTOR activity. However mTOR inhibition restored autophagy, therefore, consistent with our findings TAK1 may influence autophagy at the level of or upstream of mTOR (Inokuchi-Shimizu et al., 2014).

Experiments investigating TAK1 have highlighted a relationship between TGFβ signaling, autophagy and cancer. For example, the genetic deletion of *TAK1* blocked growth and migration of hepatocellular carcinoma (Inokuchi-Shimizu et al., 2014). Likewise, TAK1 knockdown experiments attenuated tumor growth in xenograft models (Inokuchi-Shimizu et al., 2014; Hindi et al., 2018). One possible explanation for this is that TAK1 expression is positively correlated with mTOR expression and phosphorylation. Therefore, as the activity of TAK1 increases, autophagic flux decreases and disrupts the tumor promoting properties of autophagy in cancer cells (Cheng et al., 2019). In support of this, inhibition of TAK1 in Kras-dependent NSCLC cell lines induced apoptosis by inhibiting protective autophagy (Yang et al., 2018).

TRAF6 is an E3 ubiquitin ligase proven to be essential for toll-like receptor 4-dependent autophagy. TRAF6 stabilizes beclin-1 by conjugating it to lysine(K)63-linked polyubiquitin chains (Shi and Kehrl, 2010). Furthermore, TRAF6 in partnership with autophagy and beclin-1 regulator 1 tethers ULK1 to K63-linked polyubiquitin chains to promote its stability, self-association and kinase activity (Nazio et al., 2013; Zhao and Zhang, 2016). Interestingly, TRAF6 may be a suitable therapeutic target for the pro-tumorigenic properties of autophagy. For instance, peroxiredoxin 1, an antioxidant enzyme, was observed to inhibit TRAF6 ubiquitin-ligase activity, downregulate autophagy and inhibit cancer cell migration (Min et al., 2018). Additionally, blocking TRAF6 in mice models of cancer cachexia attenuated autophagy-dependent muscle wasting (Paul and Kumar, 2011). For this reason, future work is needed to explore how silencing TRAF6 influences TGFβ-dependent autophagy and the pro-tumorigenic properties of TGFβ.

To date, P38 MAPK has been implicated in augmenting cancer cachexia by upregulating autophagy. For example, stimulating toll-like receptors in mice upregulated *ATG6*, *ATG7*, and *ATG12* expression in a P38 MAPK-dependent manner to promote muscle wasting. When P38 MAPK activity was blocked with SB202190, *ATG* genes were downregulated and mice were rescued from muscle wasting phenotypes (McClung et al., 2010). However, the role of P38 MAPK in autophagy is cell type dependent. For instance, in microglial cells, after lipopolysaccharide stimulate toll-like receptors, P38 MAPK is activated and phosphorylates ULK1, which disrupts ULK1 from recruiting ATG13 and other components of the ULK1 complex (He et al., 2018). Furthermore, another study identified that when SB202190 blocked P38 MAPK activity, the p53-dependent apoptotic response is interrupted and autophagy was upregulated, which promoted cancer cell resistance to 5-fluorouracil (De La Cruz-Morcillo et al., 2012). Recently, evidence has emerged that flavopereirine, a chemotherapeutic agent that decreases the proliferation and viability of cancer cells largely through unknown mechanisms, inhibited autophagy by upregulating the P38 MAPK pathway (Chen et al., 2020). Although these forms of autophagy are independent of TGFβ, they are still important to understanding a potential relationship between TGFβ, cancer and autophagy.

In summary, TGFβ1 regulates autophagy using Smad4 and TAK1-TRAF6-P38 MAPK pathways to influence AMPK-dependent ULK1 S555 phosphorylation. Future work will evaluate how silencing Smad4 and the TAK1-TRAF6-P38 MAPK pathway impacts pro-tumorigenic properties of TGFβ and autophagy.

## Data Availability Statement

The raw data supporting the conclusions of this article will be made available by the authors, without undue reservation.

## Author Contributions

CT carried out the work presented and finalized the writing of the manuscript. GD supervised the studies, helped design the overall experimental approach, and helped prepare the final manuscript. Both authors contributed to the article and approved the submitted version.

## Conflict of Interest

The authors declare that the research was conducted in the absence of any commercial or financial relationships that could be construed as a potential conflict of interest.

## Publisher’s Note

All claims expressed in this article are solely those of the authors and do not necessarily represent those of their affiliated organizations, or those of the publisher, the editors and the reviewers. Any product that may be evaluated in this article, or claim that may be made by its manufacturer, is not guaranteed or endorsed by the publisher.

## Supplementary Material

Supplementary Figure 1 The effect of TGFβ1 on the nuclear intensity of ULK1 and ULK2. A549 cells were treated with 250 pM TGFβ1 for 24 h. The cells were fixed and stained with DAPI (blue), anti-ULK1 (green) and anti-ULK2 (red). A Nikon Eclipse Ti2 confocal microscope was used to visualize the cells and an optical slice through the nucleus was imaged. ImageJ (version 2.0) quantified relative nuclear ULK1 intensity/Total ULK1 intensity, which are graphed below representative images (*n* = 3 ± SD). Significance is indicated as ^∗^ = *P* < 0.05 and ^****^ = *P* < 0.0001. Bar = 10 μm.

Supplementary Figure 2 The effect of a second series of ULK1 and ULK2 siRNAs on TGFβ1-dependent autophagy. A549 **(A)** or H1299 **(B)** cells stably expressing GFP-LC3-RFP-LC3ΔG were transfected with control siRNA (si-Control), siRNA targeting ULK1 (si-ULK1; s15965) or siRNA targeting ULK2 (si-ULK2; s18705) for 48 h. The cells were incubated in the absence or presence of 250 pM TGFβ1 for 24 h, lysed and subjected to SDS-PAGE and immunoblotted for anti-ULK1, anti-ULK2, anti-LC3B and anti-GAPDH antibodies. Quantitative analysis of the GFP/RFP ratios are shown graphically to the right of representative immunoblots (*n* = 3 ± SD). Significance is indicated as ^∗^ = *P* < 0.05.

Supplementary Figure 3 The effect of a second Smad4 specific siRNA on TGFβ1-dependent autophagy. A549 **(A)** or H1299 **(B)** cells stably expressing GFP-LC3-RFP-LC3ΔG were transfected with si-Control or siRNA targeting Smad4 (si-Smad4-1; s534708 or si-Smad4-2; s8404) for 48 h. The cells were incubated in the absence or presence of 250 pM TGFβ1 for 24 h, lysed and subjected to SDS-PAGE and immunoblotted for anti-Smad4, anti-LC3B and anti-GAPDH antibodies. Quantitative analysis of the GFP/RFP ratios are shown graphically to the right of representative immunoblots (*n* = 3 ± SD). Significance is indicated as ^∗^ = *P* < 0.05 and ^∗∗^ = *P* < 0.01.

Supplementary Figure 4 The effect of aPKC knockdown on TGFβ1-dependent autophagy in NSCLC cell lines. A549 **(A)** or H1299 **(B)** cells stably expressing GFP-LC3-RFP-LC3ΔG were transfected with si-Control or siRNA targeting aPKCζ (s11128) and aPKCι (s11110) for 48 h. The cells were incubated in the absence or presence of 250 pM TGFβ1 for 24 h, lysed and subjected to SDS-PAGE and immunoblotted for anti-aPKCζ, anti-aPKCι, anti-LC3B, and anti-GAPDH antibodies. Quantitative analysis of steady state LC3B-II protein levels and the GFP/RFP ratio are shown below representative immunoblots (*n* = 3 ± SD). Significance is indicated as ^∗^ = *P* < 0.05, ^∗∗^ = *P* < 0.01, and ^****^ = *P* < 0.0001. **(C)** A549 cells stably expressing GFP-LC3-RFP-LC3ΔG were treated as described above. Hoechst stain (blue) was added 10 min prior to imaging with a 63x objective using an Olympus IX 81 inverted fluorescence microscope. Bar = 10 μm. **(D)** ImageJ was used to quantify the green and red pixel intensities, and the GFP/RFP ratio is shown below representative images (*n* = 3 ± SD). Significance is indicated as ^∗∗^ = *P* < 0.01. **(E)** Cells and number of puncta/cell were counted using ImageJ version 2.0 software. The data were graphed and shown below representative images (*n* = 3 ± SD). Significance is indicated as ^∗∗^ = *P* < 0.01.

Supplementary Figure 5 The effect of P38 MAPK on TGFβ1 induced autophagy in NSCLC cell lines. A549 **(A)** or H1299 **(B)** cells stably expressing GFP-LC3-RFP-LC3ΔG were treated with 10 μM P38 MAPK or equivalent volumes of DMSO in the presence and absence of 250 pM TGFβ1 for 24 h. Cells were lysed and subjected to SDS-PAGE and immunoblotting for anti-cleaved parp, anti-LC3B and anti-GAPDH antibodies. Quantitative analysis of steady state ULK1 and LC3B-II protein levels and the GFP/RFP ratio are shown graphically below representative immunoblots (*n* = 3 ± SD). Significance is indicated as ^∗^ = *P* < 0.05, ^∗∗^ = *P* < 0.01, and ^∗∗∗^ = *P* < 0.001. **(C)** A549 cells stably expressing a cDNA GFP-LC3-RFP-LC3ΔG construct were treated as described above. Hoechst stain (blue) was added 10 min prior to imaging with a 63x objective using an Olympus IX 81 inverted fluorescence microscope. Bar = 10 μm. **(D)** ImageJ quantified the green and red pixel intensity, and the GFP/RFP ratio is shown graphically below representative images (*n* = 3 ± SD). Significance is indicated as ^∗^ = *P* < 0.05. **(E)** Cells and number of puncta/cell were counted using ImageJ version 2.0 software. The data were graphed and shown graphically below representative images (*n* = 3 ± SD). Significance is indicated as ^∗∗^ = *P* < 0.01.

Supplementary Figure 6 The effect of TRAF6 silencing on TGFβ1-dependent autophagy in NSCLC cell lines. A549 **(A)** or H1299 **(B)** cells stably expressing GFP-LC3-RFP-LC3ΔG were transfected with si-Control or si-TRAF6 (s14388) for 48 h. The cells were incubated in the absence or presence of 250 pM TGFβ1 for 24 h, lysed and subjected to SDS-PAGE and immunoblotted for anti-TRAF6, anti-LC3B and anti-GAPDH antibodies. Quantitative analysis of steady state LC3B-II protein levels and the GFP/RFP ratio are shown graphically below representative immunoblots (*n* = 3 ± SD). Significance is indicated as ^∗^ = *P* < 0.05, ^∗∗^ = *P* < 0.01, and ^****^ = *P* < 0.0001. **(C)** A549 cells stably expressing a cDNA GFP-LC3-RFP-LC3ΔG construct were treated as described above. Hoechst stain (blue) was added 10 min prior to imaging with a 63x objective using an Olympus IX 81 inverted fluorescence microscope. Bar = 10 μm. **(D)** ImageJ quantified the green and red pixel intensity, and the GFP/RFP ratio is shown graphically below representative images (*n* = 3 ± SD). Significance is indicated as ^∗^ = *P* < 0.05. **(E)** Cells and number of puncta/cell were counted using ImageJ version 2.0 software. The data were graphed and shown graphically below representative images (*n* = 3 ± SD). Significance is indicated as ^∗∗∗^ = *P* < 0.001.

Supplementary Figure 7 The effect of TAK1 silencing on TGFβ1-dependent autophagy in NSCLC cell lines. A549 **(A)** or H1299 **(B)** cells stably expressing GFP-LC3-RFP-LC3ΔG were transfected with si-Control or si-TAK1 (s13766) for 48 h. The cells were incubated in the absence or presence of 250 pM TGFβ1 for 24 h, lysed and subjected to SDS-PAGE and immunoblotted for anti-TAK1, anti-LC3B and anti-GAPDH antibodies. Quantitative analysis of steady state LC3B-II protein levels and the GFP/RFP ratio are shown graphically below representative immunoblots (*n* = 3 ± SD). Significance is indicated as ^∗^ = *P* < 0.05, ^∗∗^ = *P* < 0.01, and ^∗∗∗^ = *P* < 0.001. **(C)** A549 cells stably expressing a cDNA GFP-LC3-RFP-LC3ΔG construct were treated as described above. Hoechst stain (blue) was added 10 min prior to imaging with a 63x objective using an Olympus IX 81 inverted fluorescence microscope. Bar = 10 μm. **(D)** ImageJ quantified the green and red pixel intensity, and the GFP/RFP ratio is shown graphically below representative images (*n* = 3 ± SD). Significance is indicated as ^∗^ = *P* < 0.05. **(E)** Cells and number of puncta/cell were counted using ImageJ version 2.0 software. The data were graphed and shown graphically below representative images (*n* = 3 ± SD). Significance is indicated as ^∗∗∗^ = *P* < 0.001 and ^****^ = *P* < 0.0001.

Supplementary Figure 8 The influence of a second TAK1 and TRAF6 series of siRNAs in combination with a P38 MAPK inhibitor on TGFβ1-dependent autophagy. A549 **(A)** or H1299 **(B)** cells stably expressing GFP-LC3-RFP-LC3ΔG were transfected with si-Control or si-TRAF6 (s14389) for 48 h. The cells were incubated in the absence or presence of 250 pM TGFβ1 for 24 h, lysed and subjected to SDS-PAGE and immunoblotted for anti-TRAF6, anti-LC3B and anti-GAPDH antibodies. Quantitative analysis of the GFP/RFP ratios are shown graphically to the right of representative immunoblots (*n* = 3 ± SD). Significance is indicated as ^∗^ = *P* < 0.05 and ^****^ = *P* < 0.0001. A549 **(C)** or H1299 **(D)** cells stably expressing GFP-LC3-RFP-LC3ΔG were transfected with si-Control or si-TAK1 (s13767) for 48 h. The cells were incubated in the absence or presence of 250 pM TGFβ1 for 24 h, lysed and subjected to SDS-PAGE and immunoblotted for anti-TAK1, anti-LC3B and anti-GAPDH antibodies. Quantitative analysis of the GFP/RFP ratios are shown graphically to the right of representative immunoblots (*n* = 3 ± SD). Significance is indicated as ^∗^ = *P* < 0.05. A549 **(E)** or H1299 **(F)** cells stably expressing GFP-LC3-RFP-LC3ΔG were transfected with si-Control or si-TAK1 (s13767) and si-TRAF6 (s14389) for 48 h. The cells were incubated in the absence or presence of 250 pM TGFβ1 and 10 μM P38 MAPK inhibitor for 24 h. The cells were then lysed, subjected to SDS-PAGE and immunoblotted for anti-TAK1, anti-TRAF6, anti-cleaved PARP, anti-LC3B and anti-GAPDH antibodies. Quantitative analysis of GFP/RFP ratios are shown to the right of representative immunoblots (*n* = 3 ± SD). Significance is indicated as ^∗^ = *P* < 0.05 and ^∗∗^ = *P* < 0.01.

Supplementary Figure 9 The effect of Compound C on TGFβ1 induced autophagy in NSCLC cell lines. **(A)** A549 cells stably expressing GFP-LC3-RFP-LC3ΔG were treated with 10 μM Compound C or DMSO (vehicle control) in the presence and absence of 250 pM TGFβ1 for 24 h. Cells were lysed and subjected to SDS-PAGE and immunoblotting for anti-ULK1, anti-phospho-S555-ULK1, anti-AMPKα, anti-phospho-T172-AMPKα anti-LC3B and anti-GAPDH antibodies. Quantitative analysis of steady state LC3B-II protein levels and the GFP/RFP, P-AMPKα/AMPKα, P-ULK1/ULK1 ratios are shown below representative immunoblots (*n* = 3 ± SD). Significance is indicated as ^∗^ = *P* < 0.05, ^∗∗^ = *P* < 0.01, ^∗∗∗^ = *P* < 0.001, and ^****^ = *P* < 0.0001. **(B)** A549 cells stably expressing a cDNA GFP-LC3-RFP-LC3ΔG construct were treated as described above. Hoechst stain (blue) was added 10 min prior to imaging with a 63x objective using an Olympus IX 81 inverted fluorescence microscope. Bar = 10 μm. ImageJ was used to quantify the green and red pixel intensities, and the GFP/RFP ratio is shown below representative images (*n* = 3 ± SD). Significance is indicated as ^∗∗^ = *P* < 0.01, ^∗∗∗^ = *P* < 0.001, and ^****^ = *P* < 0.0001.

Supplementary Figure 10 The effect of canonical and non-canonical TGFβ signaling on the cellular distribution of ULK1. **(A)** A549 cells were transfected with si-Control or two different siRNAs targeting Smad4 (si-Smad4-1; s534708 and siSmad4-2; s8404) for 48 h. The cells were incubated in the absence or presence of 250 pM TGFβ1 for 24 h, fixed and stained with DAPI (blue) or anti-ULK1 (green). A Nikon Eclipse Ti2 confocal microscope was used to visualize the cells and an optical slice through the nucleus was imaged. ImageJ (version 2.0) quantified relative nuclear ULK1 intensity/Total ULK1 intensity, which are graphed below representative images (*n* = 3 ± SD). Significance is indicated as ^∗∗^ = *P* < 0.01, ^∗∗∗^ = *P* < 0.001, and ^****^ = *P* < 0.0001. Bar = 10 μm. **(B)** A549 cells were transfected with si-Control or two different siRNAs against TAK1 (si-TAK1-1; s13766 and TAK1-2; s13767) or TRAF6 (si-TRAF6-1; s14388 and TRAF6-2; s14389) for 48 h. The cells were incubated in the absence or presence of 250 pM TGFβ1 and 10 μM P38 MAPK inhibitor for 24 h. The cells were then fixed and stained with DAPI (blue) or anti-ULK1 (green). A Nikon Eclipse Ti2 confocal microscope was used to visualize the cells and an optical slice through the nucleus was imaged. ImageJ (version 2.0) quantified relative nuclear ULK1 intensity/Total ULK1 intensity, which are graphed below representative images (*n* = 3 ± SD). Significance is indicated as ^∗^ = *P* < 0.05 and ^∗∗^ = *P* < 0.01. Bar = 10 μm.

## Abbreviations

AMPK: 5 ′ adenosine monophosphate-activated protein kinase
aPKC: atypical protein kinase C
ATG: autophagy related
DMSO: dimethyl sulfoxide
EMT: epithelial-mesenchymal transition
F-12K: Kaighn’s modification of Hams F-12
FBS: fetal bovine serum
GFP: green fluorescent protein
HRP: horseradish-peroxidase
K: lysine
LC3: microtubule-associated membrane protein light-chain 3
NSCLC: non-small cell lung cancer
MAPK: mitogen-activated protein kinase
mTOR: mechanistic target of rapamycin
PI3K: phosphoinositide-3 kinase
P-Smad 2: phosphorylated Smad2
R-Smad: receptor-Smad
RFP: red fluorescent protein
RPMI: Roswell Park Memorial Institute
SARA: smad anchor for receptor activation
S: serine
SDS-PADE: sodium dodecyl sulfate polyacrylamide gel electrophoresis
siRNA: small interfering RNA
TAK1: TGF β-activated kinase 1
TGF β 1: transforming growth factor beta 1
TGF β R: transforming growth factor beta receptor
TRAF6: tumor necrosis factor receptor-associated factor 6
ULK: unc 51-like kinase.
