# Supplementary figures and images for: Canonical and Non-canonical TGFβ Signaling Activate Autophagy in an ULK1-Dependent Manner
Source: Front Cell Dev Biol. 2021 Oct 25;9:712124. doi: 10.3389/fcell.2021.712124 (PMC8573198; doi:10.3389/fcell.2021.712124)

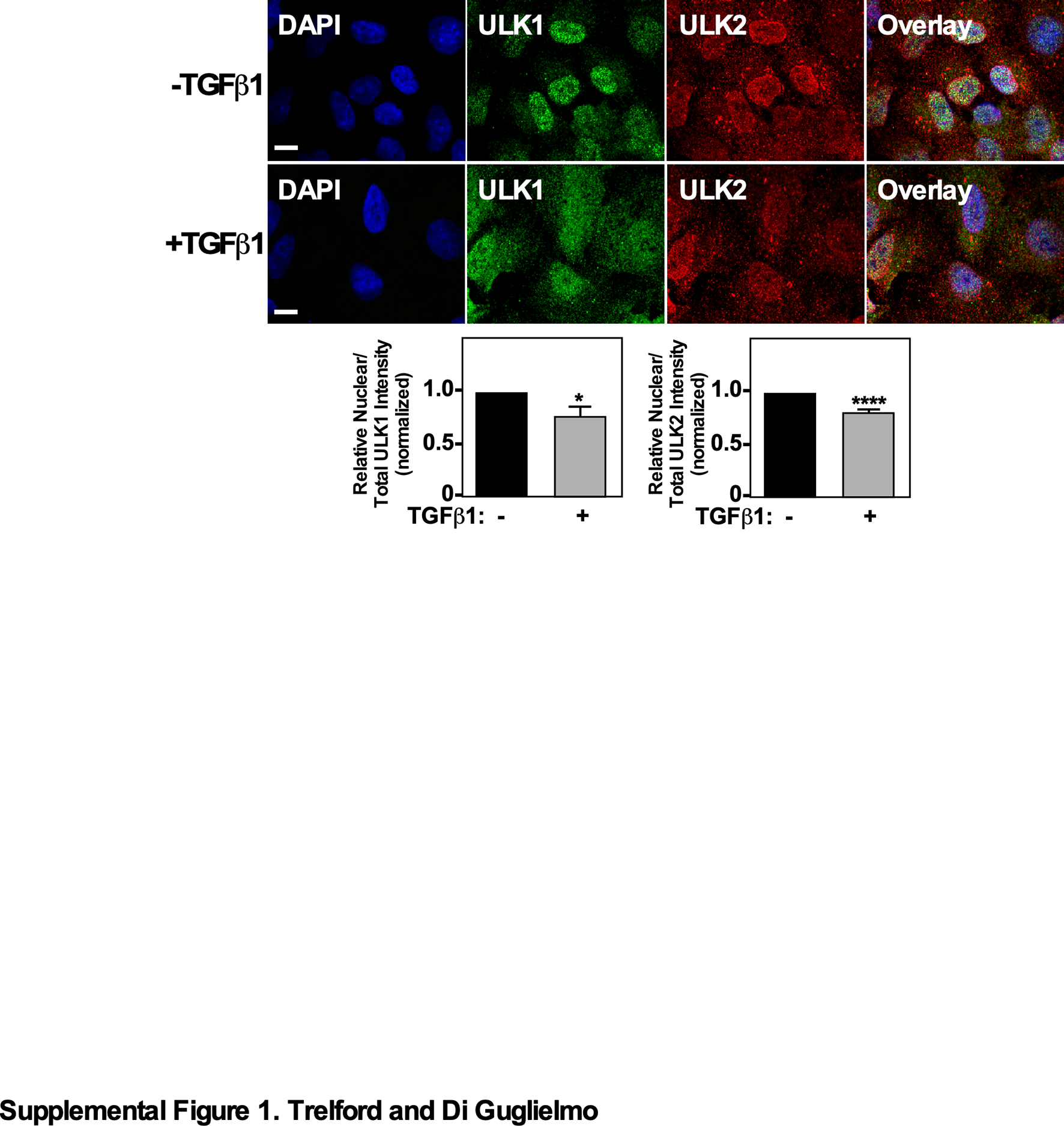

Supplement: Supplementary Figure 1 — The effect of TGFβ1 on the nuclear intensity of ULK1 and ULK2. A549 cells were treated with 250 pM TGFβ1 for 24 h. The cells were fixed and stained with DAPI (blue), anti-ULK1 (green) and anti-ULK2 (red). A Nikon Eclipse Ti2 confocal microscope was used to visualize the cells and an optical slice through the nucleus was imaged. ImageJ (version 2.0) quantified relative nuclear ULK1 intensity/Total ULK1 intensity, which are graphed below representative images (n = 3 ± SD). Significance is indicated as ∗ = P < 0.05 and **** = P < 0.0001. Bar = 10 μm. [file Image_1.TIF]

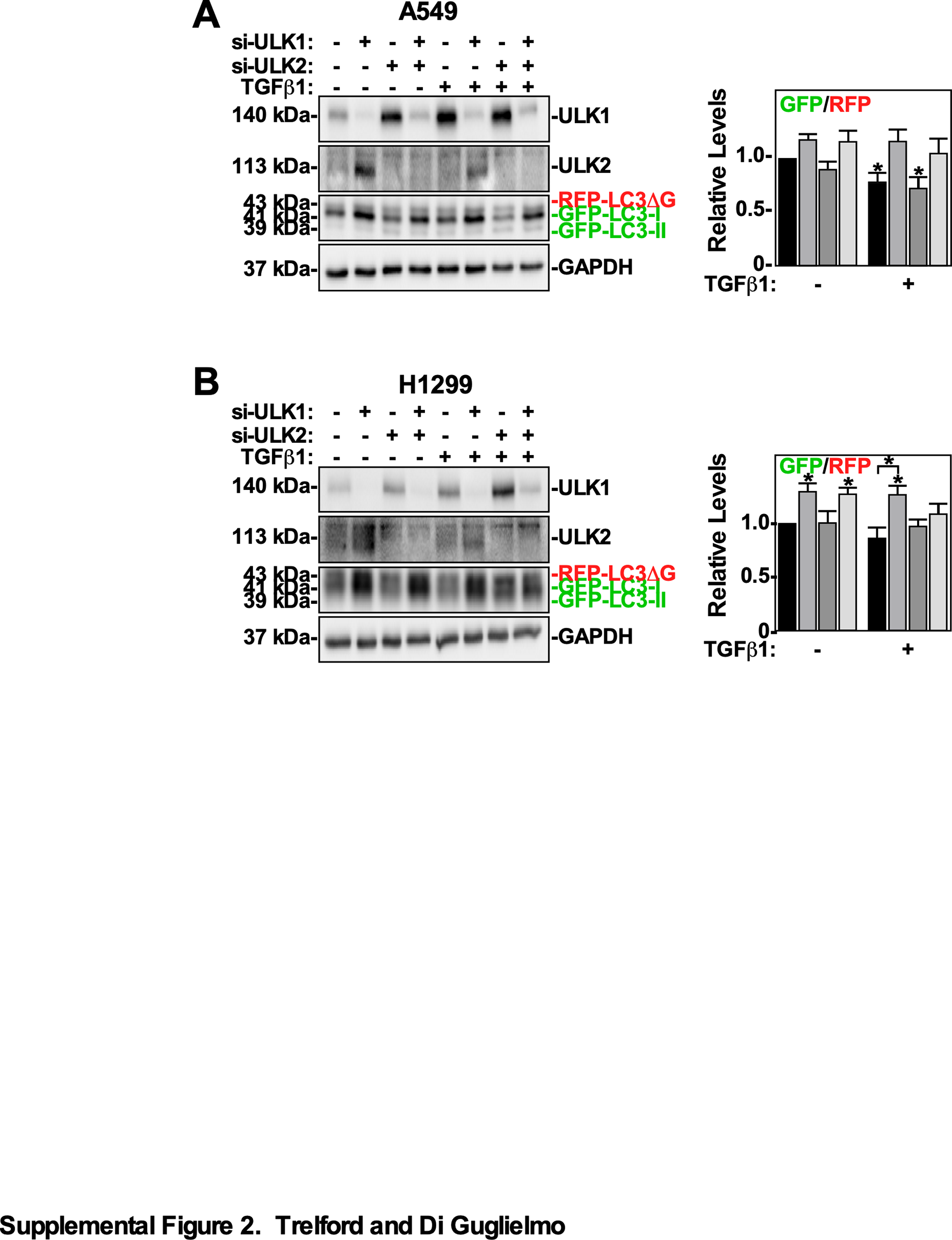

Supplement: Supplementary Figure 2 — The effect of a second series of ULK1 and ULK2 siRNAs on TGFβ1-dependent autophagy. A549 (A) or H1299 (B) cells stably expressing GFP-LC3-RFP-LC3ΔG were transfected with control siRNA (si-Control), siRNA targeting ULK1 (si-ULK1; s15965) or siRNA targeting ULK2 (si-ULK2; s18705) for 48 h. The cells were incubated in the absence or presence of 250 pM TGFβ1 for 24 h, lysed and subjected to SDS-PAGE and immunoblotted for anti-ULK1, anti-ULK2, anti-LC3B and anti-GAPDH antibodies. Quantitative analysis of the GFP/RFP ratios are shown graphically to the right of representative immunoblots (n = 3 ± SD). Significance is indicated as ∗ = P < 0.05. [file Image_2.TIF]

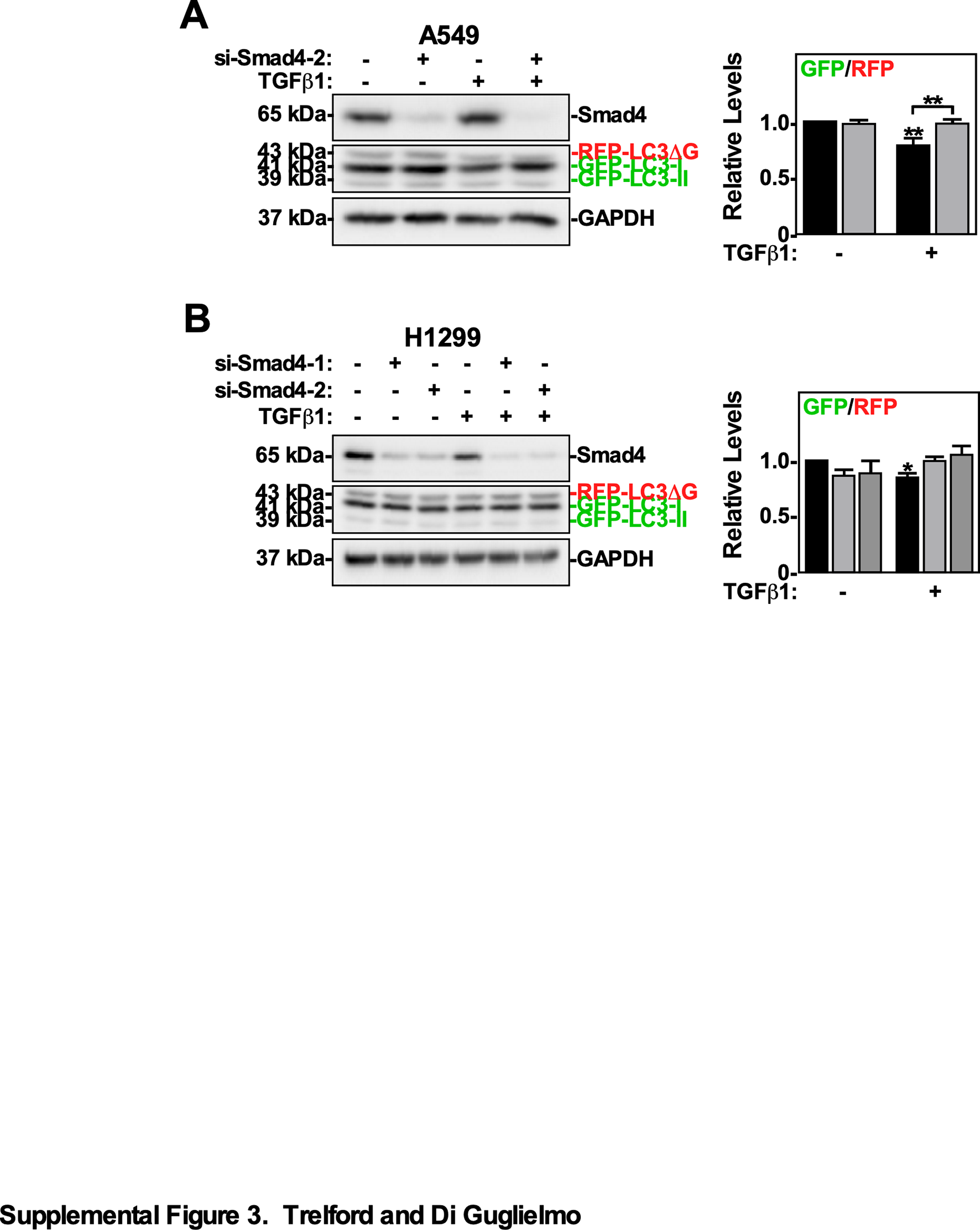

Supplement: Supplementary Figure 3 — The effect of a second Smad4 specific siRNA on TGFβ1-dependent autophagy. A549 (A) or H1299 (B) cells stably expressing GFP-LC3-RFP-LC3ΔG were transfected with si-Control or siRNA targeting Smad4 (si-Smad4-1; s534708 or si-Smad4-2; s8404) for 48 h. The cells were incubated in the absence or presence of 250 pM TGFβ1 for 24 h, lysed and subjected to SDS-PAGE and immunoblotted for anti-Smad4, anti-LC3B and anti-GAPDH antibodies. Quantitative analysis of the GFP/RFP ratios are shown graphically to the right of representative immunoblots (n = 3 ± SD). Significance is indicated as ∗ = P < 0.05 and ∗∗ = P < 0.01. [file Image_3.TIF]

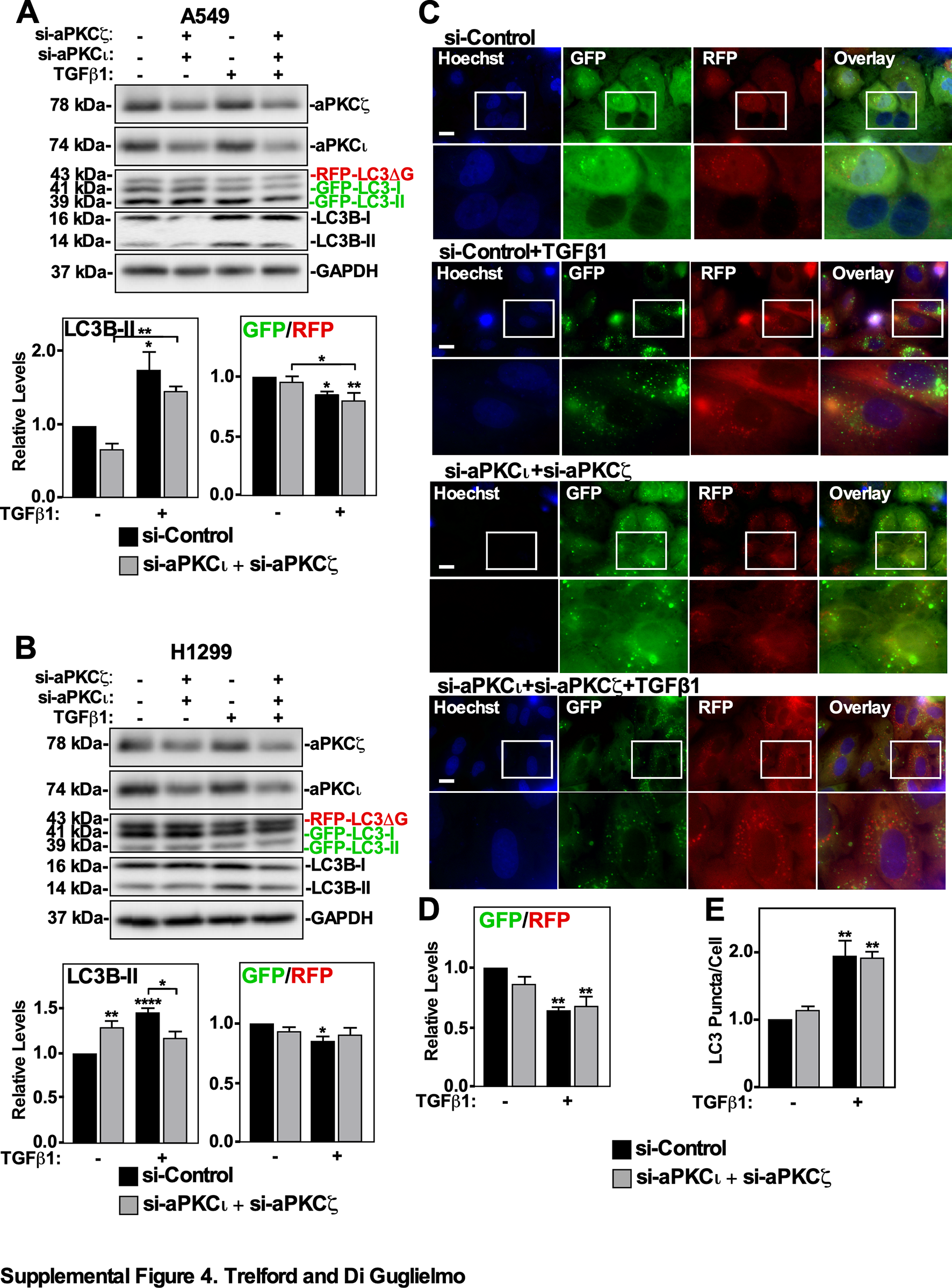

Supplement: Supplementary Figure 4 — The effect of aPKC knockdown on TGFβ1-dependent autophagy in NSCLC cell lines. A549 (A) or H1299 (B) cells stably expressing GFP-LC3-RFP-LC3ΔG were transfected with si-Control or siRNA targeting aPKCζ (s11128) and aPKCι (s11110) for 48 h. The cells were incubated in the absence or presence of 250 pM TGFβ1 for 24 h, lysed and subjected to SDS-PAGE and immunoblotted for anti-aPKCζ, anti-aPKCι, anti-LC3B, and anti-GAPDH antibodies. Quantitative analysis of steady state LC3B-II protein levels and the GFP/RFP ratio are shown below representative immunoblots (n = 3 ± SD). Significance is indicated as ∗ = P < 0.05, ∗∗ = P < 0.01, and **** = P < 0.0001. (C) A549 cells stably expressing GFP-LC3-RFP-LC3ΔG were treated as described above. Hoechst stain (blue) was added 10 min prior to imaging with a 63x objective using an Olympus IX 81 inverted fluorescence microscope. Bar = 10 μm. (D) ImageJ was used to quantify the green and red pixel intensities, and the GFP/RFP ratio is shown below representative images (n = 3 ± SD). Significance is indicated as ∗∗ = P < 0.01. (E) Cells and number of puncta/cell were counted using ImageJ version 2.0 software. The data were graphed and shown below representative images (n = 3 ± SD). Significance is indicated as ∗∗ = P < 0.01. [file Image_4.TIF]

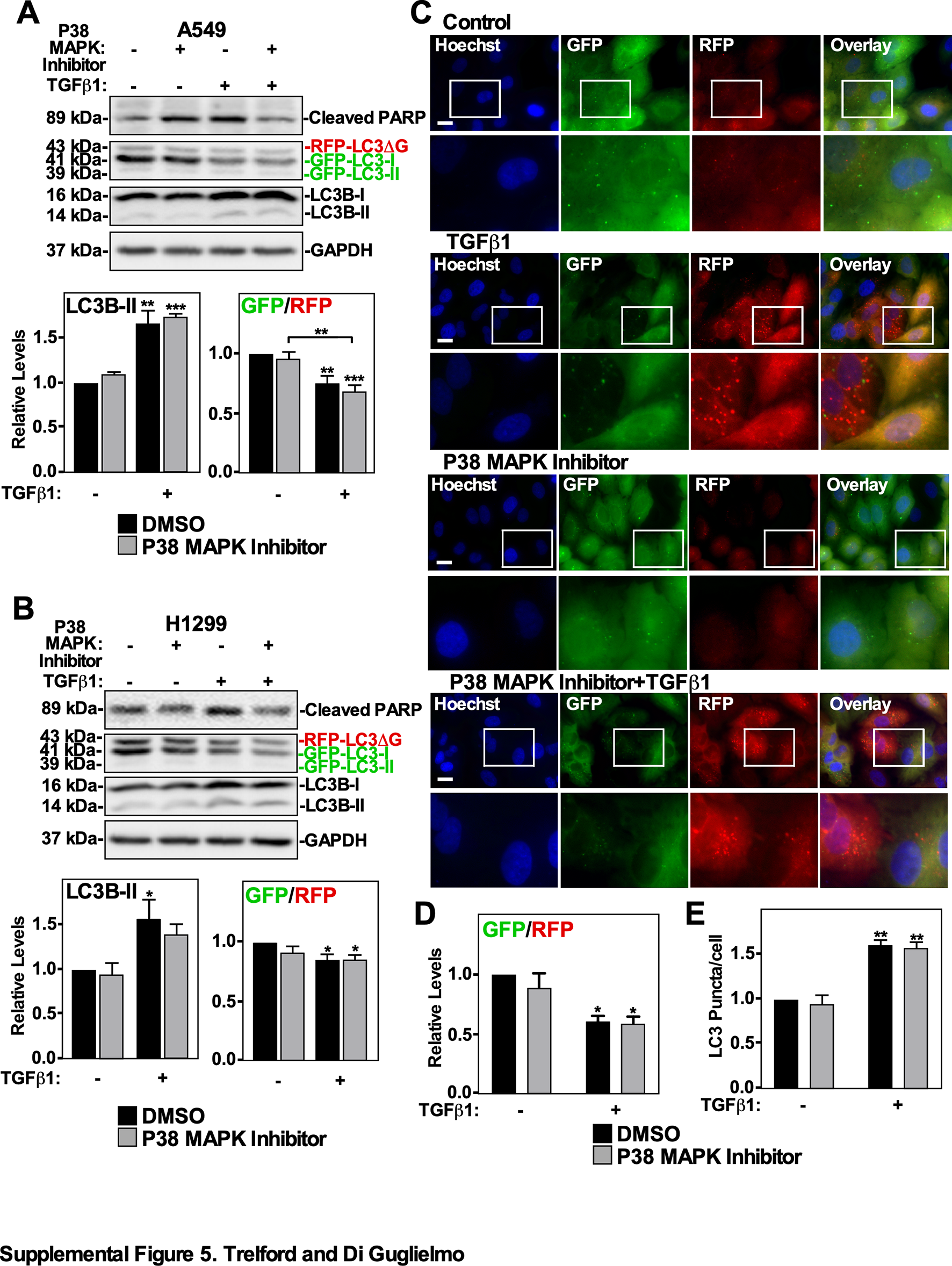

Supplement: Supplementary Figure 5 — The effect of P38 MAPK on TGFβ1 induced autophagy in NSCLC cell lines. A549 (A) or H1299 (B) cells stably expressing GFP-LC3-RFP-LC3ΔG were treated with 10 μM P38 MAPK or equivalent volumes of DMSO in the presence and absence of 250 pM TGFβ1 for 24 h. Cells were lysed and subjected to SDS-PAGE and immunoblotting for anti-cleaved parp, anti-LC3B and anti-GAPDH antibodies. Quantitative analysis of steady state ULK1 and LC3B-II protein levels and the GFP/RFP ratio are shown graphically below representative immunoblots (n = 3 ± SD). Significance is indicated as ∗ = P < 0.05, ∗∗ = P < 0.01, and ∗∗∗ = P < 0.001. (C) A549 cells stably expressing a cDNA GFP-LC3-RFP-LC3ΔG construct were treated as described above. Hoechst stain (blue) was added 10 min prior to imaging with a 63x objective using an Olympus IX 81 inverted fluorescence microscope. Bar = 10 μm. (D) ImageJ quantified the green and red pixel intensity, and the GFP/RFP ratio is shown graphically below representative images (n = 3 ± SD). Significance is indicated as ∗ = P < 0.05. (E) Cells and number of puncta/cell were counted using ImageJ version 2.0 software. The data were graphed and shown graphically below representative images (n = 3 ± SD). Significance is indicated as ∗∗ = P < 0.01. [file Image_5.TIF]

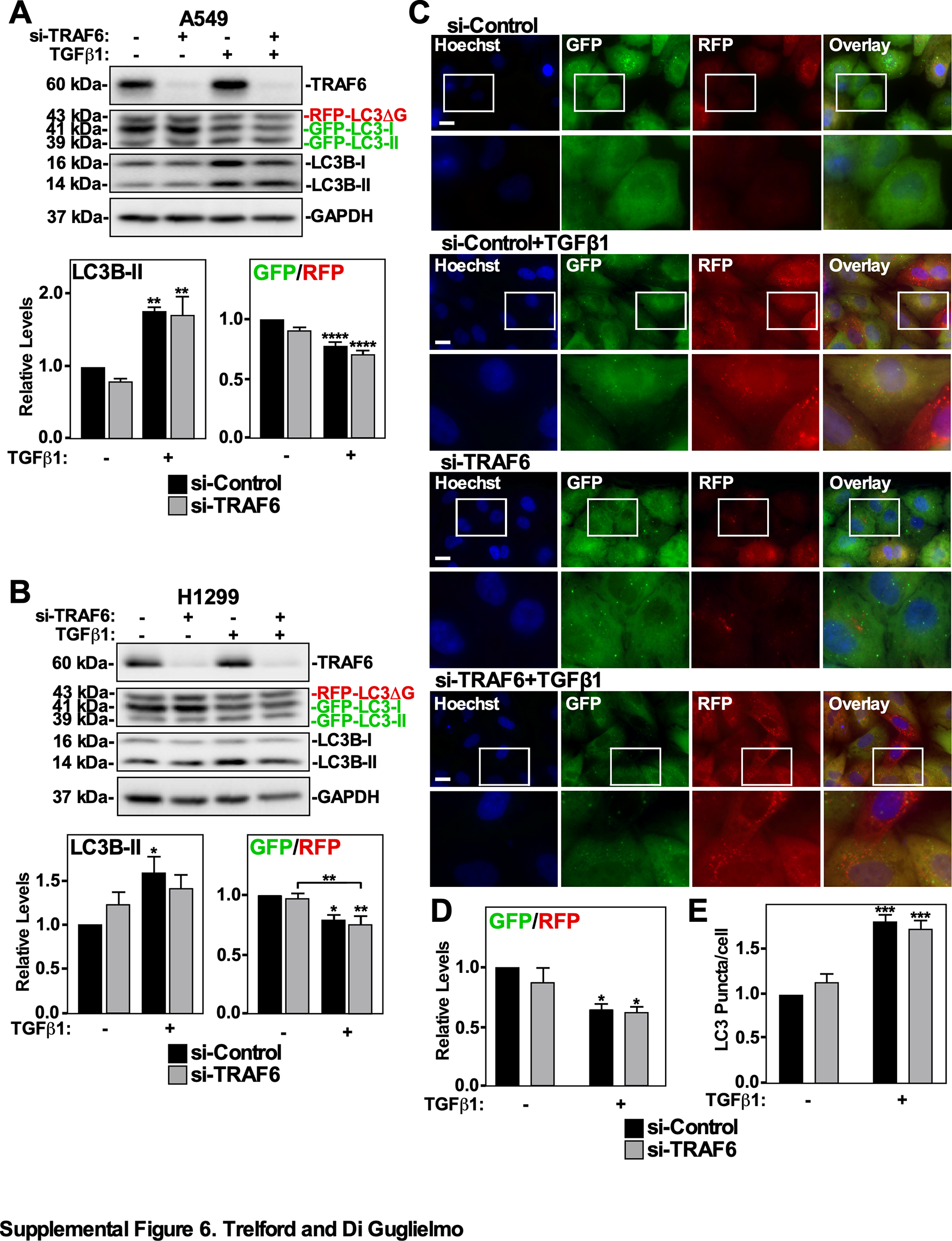

Supplement: Supplementary Figure 6 — The effect of TRAF6 silencing on TGFβ1-dependent autophagy in NSCLC cell lines. A549 (A) or H1299 (B) cells stably expressing GFP-LC3-RFP-LC3ΔG were transfected with si-Control or si-TRAF6 (s14388) for 48 h. The cells were incubated in the absence or presence of 250 pM TGFβ1 for 24 h, lysed and subjected to SDS-PAGE and immunoblotted for anti-TRAF6, anti-LC3B and anti-GAPDH antibodies. Quantitative analysis of steady state LC3B-II protein levels and the GFP/RFP ratio are shown graphically below representative immunoblots (n = 3 ± SD). Significance is indicated as ∗ = P < 0.05, ∗∗ = P < 0.01, and **** = P < 0.0001. (C) A549 cells stably expressing a cDNA GFP-LC3-RFP-LC3ΔG construct were treated as described above. Hoechst stain (blue) was added 10 min prior to imaging with a 63x objective using an Olympus IX 81 inverted fluorescence microscope. Bar = 10 μm. (D) ImageJ quantified the green and red pixel intensity, and the GFP/RFP ratio is shown graphically below representative images (n = 3 ± SD). Significance is indicated as ∗ = P < 0.05. (E) Cells and number of puncta/cell were counted using ImageJ version 2.0 software. The data were graphed and shown graphically below representative images (n = 3 ± SD). Significance is indicated as ∗∗∗ = P < 0.001. [file Image_6.TIF]

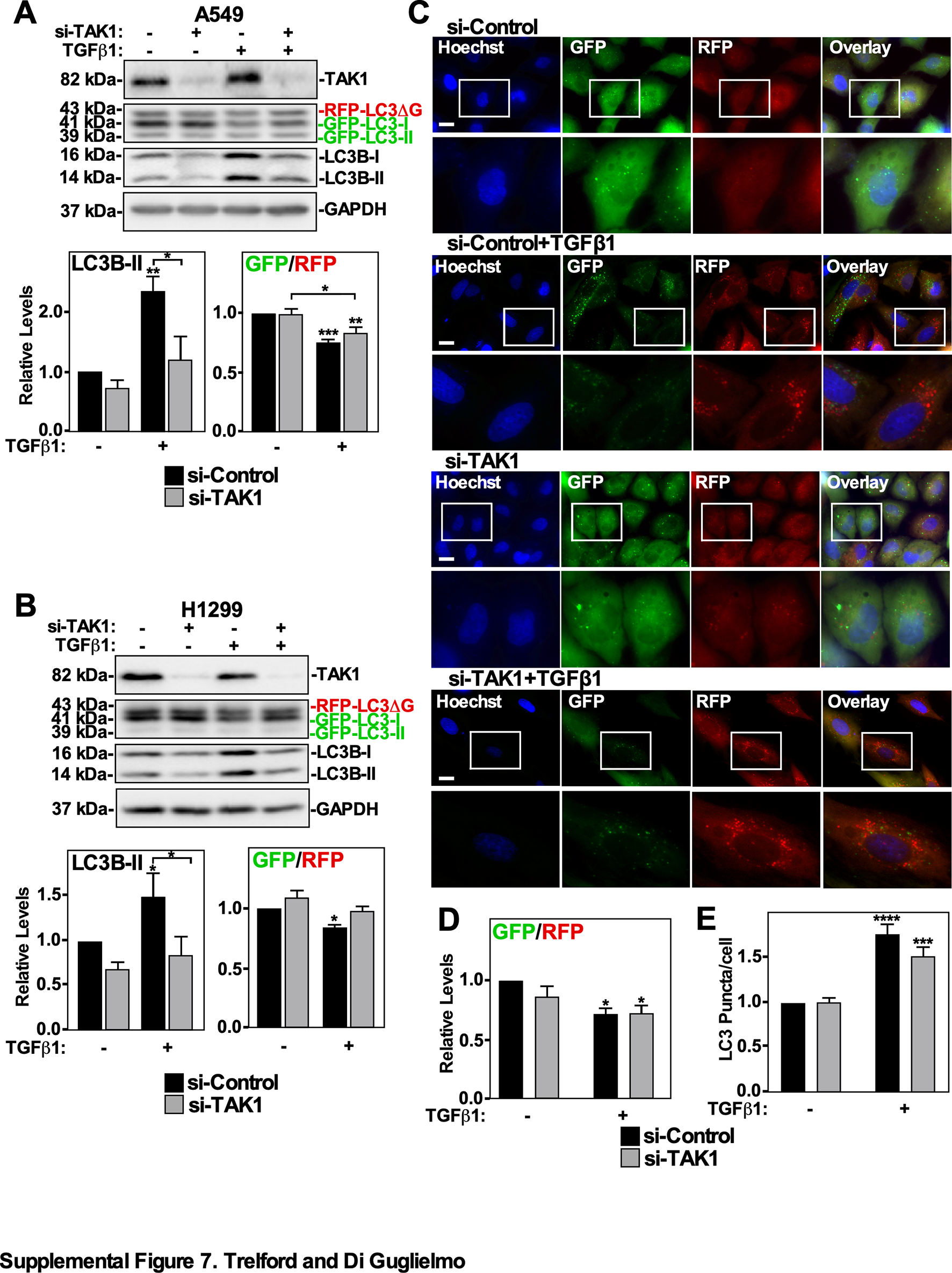

Supplement: Supplementary Figure 7 — The effect of TAK1 silencing on TGFβ1-dependent autophagy in NSCLC cell lines. A549 (A) or H1299 (B) cells stably expressing GFP-LC3-RFP-LC3ΔG were transfected with si-Control or si-TAK1 (s13766) for 48 h. The cells were incubated in the absence or presence of 250 pM TGFβ1 for 24 h, lysed and subjected to SDS-PAGE and immunoblotted for anti-TAK1, anti-LC3B and anti-GAPDH antibodies. Quantitative analysis of steady state LC3B-II protein levels and the GFP/RFP ratio are shown graphically below representative immunoblots (n = 3 ± SD). Significance is indicated as ∗ = P < 0.05, ∗∗ = P < 0.01, and ∗∗∗ = P < 0.001. (C) A549 cells stably expressing a cDNA GFP-LC3-RFP-LC3ΔG construct were treated as described above. Hoechst stain (blue) was added 10 min prior to imaging with a 63x objective using an Olympus IX 81 inverted fluorescence microscope. Bar = 10 μm. (D) ImageJ quantified the green and red pixel intensity, and the GFP/RFP ratio is shown graphically below representative images (n = 3 ± SD). Significance is indicated as ∗ = P < 0.05. (E) Cells and number of puncta/cell were counted using ImageJ version 2.0 software. The data were graphed and shown graphically below representative images (n = 3 ± SD). Significance is indicated as ∗∗∗ = P < 0.001 and **** = P < 0.0001. [file Image_7.TIF]

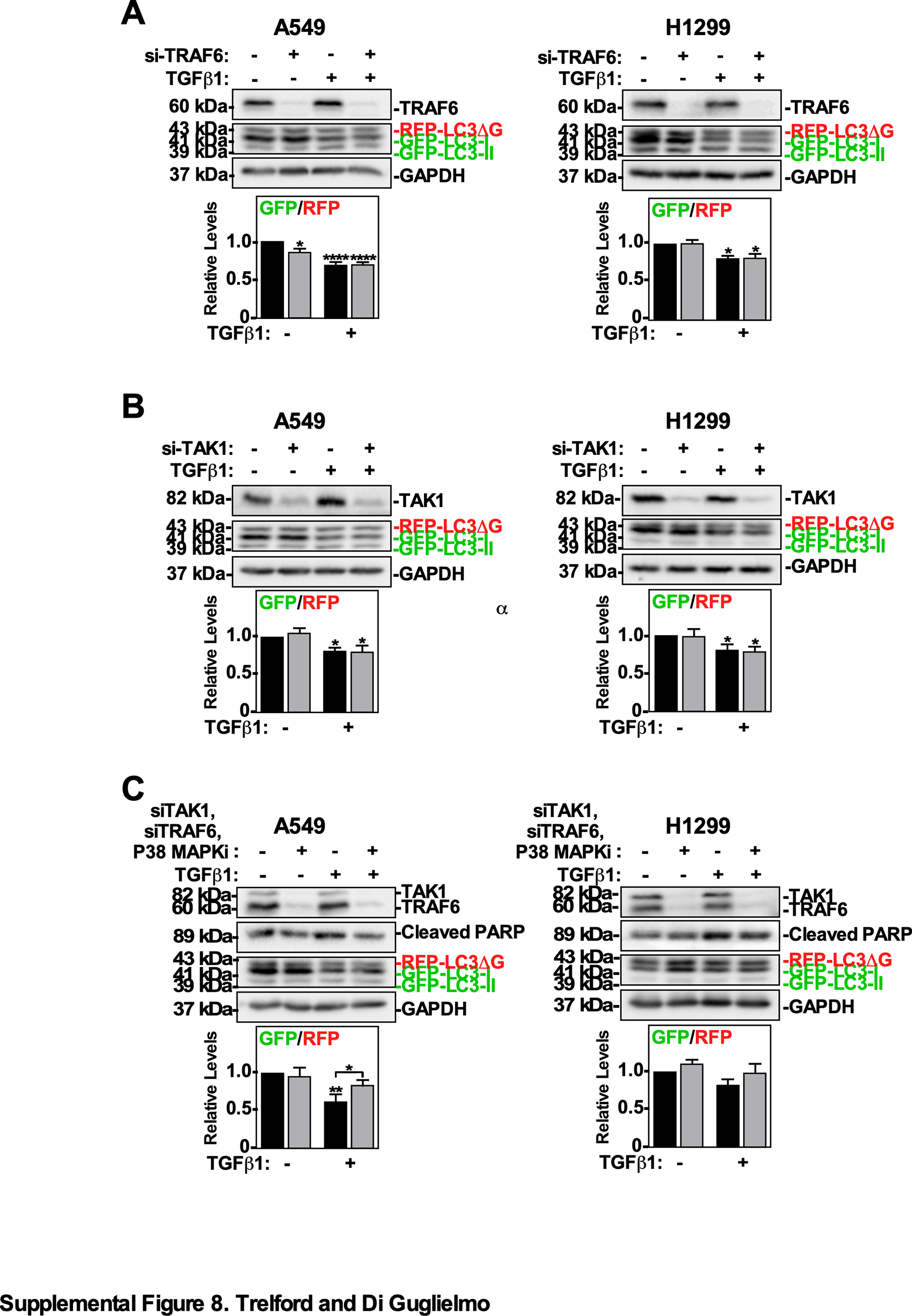

Supplement: Supplementary Figure 8 — The influence of a second TAK1 and TRAF6 series of siRNAs in combination with a P38 MAPK inhibitor on TGFβ1-dependent autophagy. A549 (A) or H1299 (B) cells stably expressing GFP-LC3-RFP-LC3ΔG were transfected with si-Control or si-TRAF6 (s14389) for 48 h. The cells were incubated in the absence or presence of 250 pM TGFβ1 for 24 h, lysed and subjected to SDS-PAGE and immunoblotted for anti-TRAF6, anti-LC3B and anti-GAPDH antibodies. Quantitative analysis of the GFP/RFP ratios are shown graphically to the right of representative immunoblots (n = 3 ± SD). Significance is indicated as ∗ = P < 0.05 and **** = P < 0.0001. A549 (C) or H1299 (D) cells stably expressing GFP-LC3-RFP-LC3ΔG were transfected with si-Control or si-TAK1 (s13767) for 48 h. The cells were incubated in the absence or presence of 250 pM TGFβ1 for 24 h, lysed and subjected to SDS-PAGE and immunoblotted for anti-TAK1, anti-LC3B and anti-GAPDH antibodies. Quantitative analysis of the GFP/RFP ratios are shown graphically to the right of representative immunoblots (n = 3 ± SD). Significance is indicated as ∗ = P < 0.05. A549 (E) or H1299 (F) cells stably expressing GFP-LC3-RFP-LC3ΔG were transfected with si-Control or si-TAK1 (s13767) and si-TRAF6 (s14389) for 48 h. The cells were incubated in the absence or presence of 250 pM TGFβ1 and 10 μM P38 MAPK inhibitor for 24 h. The cells were then lysed, subjected to SDS-PAGE and immunoblotted for anti-TAK1, anti-TRAF6, anti-cleaved PARP, anti-LC3B and anti-GAPDH antibodies. Quantitative analysis of GFP/RFP ratios are shown to the right of representative immunoblots (n = 3 ± SD). Significance is indicated as ∗ = P < 0.05 and ∗∗ = P < 0.01. [file Image_8.TIF]

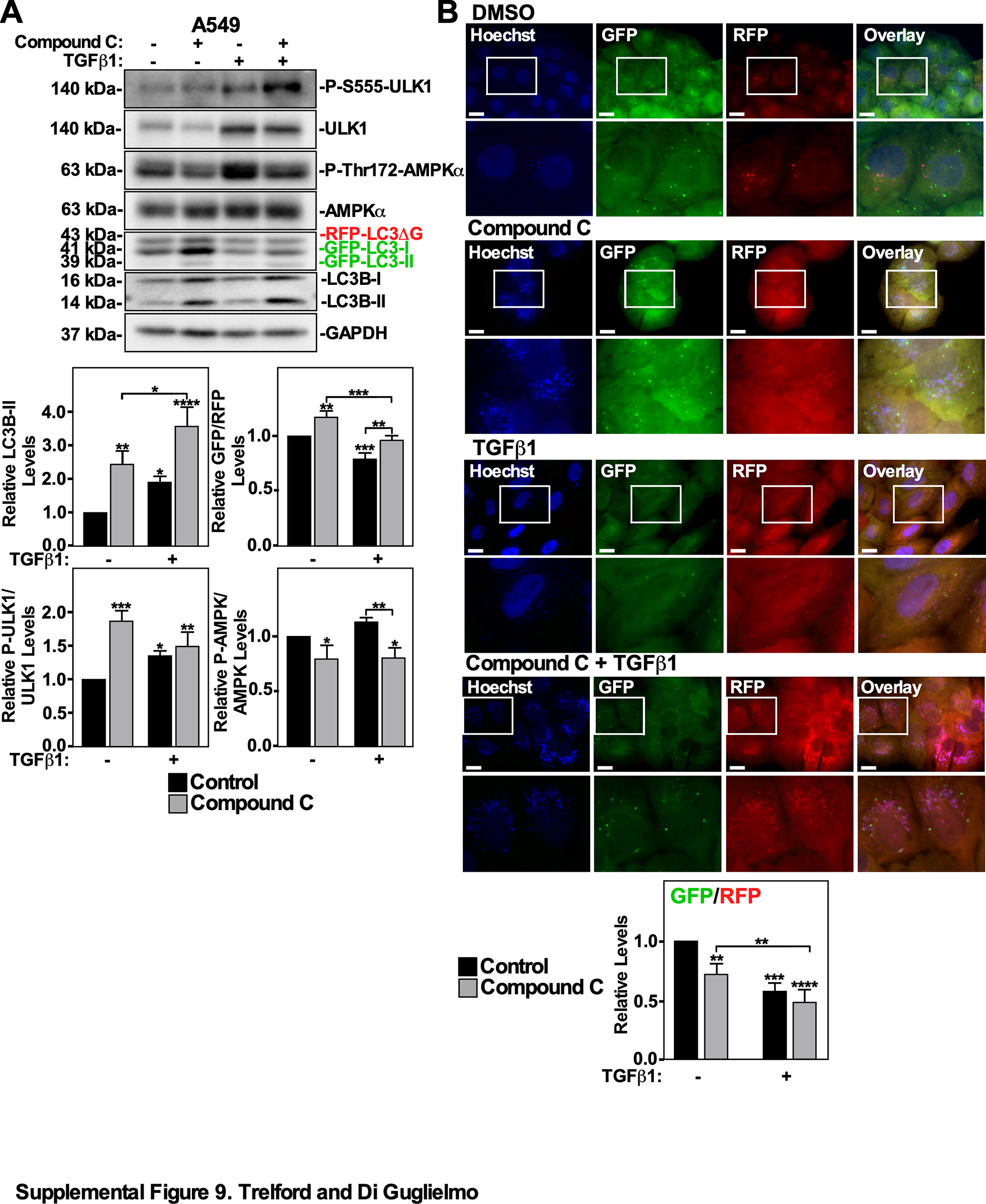

Supplement: Supplementary Figure 9 — The effect of Compound C on TGFβ1 induced autophagy in NSCLC cell lines. (A) A549 cells stably expressing GFP-LC3-RFP-LC3ΔG were treated with 10 μM Compound C or DMSO (vehicle control) in the presence and absence of 250 pM TGFβ1 for 24 h. Cells were lysed and subjected to SDS-PAGE and immunoblotting for anti-ULK1, anti-phospho-S555-ULK1, anti-AMPKα, anti-phospho-T172-AMPKα anti-LC3B and anti-GAPDH antibodies. Quantitative analysis of steady state LC3B-II protein levels and the GFP/RFP, P-AMPKα/AMPKα, P-ULK1/ULK1 ratios are shown below representative immunoblots (n = 3 ± SD). Significance is indicated as ∗ = P < 0.05, ∗∗ = P < 0.01, ∗∗∗ = P < 0.001, and **** = P < 0.0001. (B) A549 cells stably expressing a cDNA GFP-LC3-RFP-LC3ΔG construct were treated as described above. Hoechst stain (blue) was added 10 min prior to imaging with a 63x objective using an Olympus IX 81 inverted fluorescence microscope. Bar = 10 μm. ImageJ was used to quantify the green and red pixel intensities, and the GFP/RFP ratio is shown below representative images (n = 3 ± SD). Significance is indicated as ∗∗ = P < 0.01, ∗∗∗ = P < 0.001, and **** = P < 0.0001. [file Image_9.TIF]

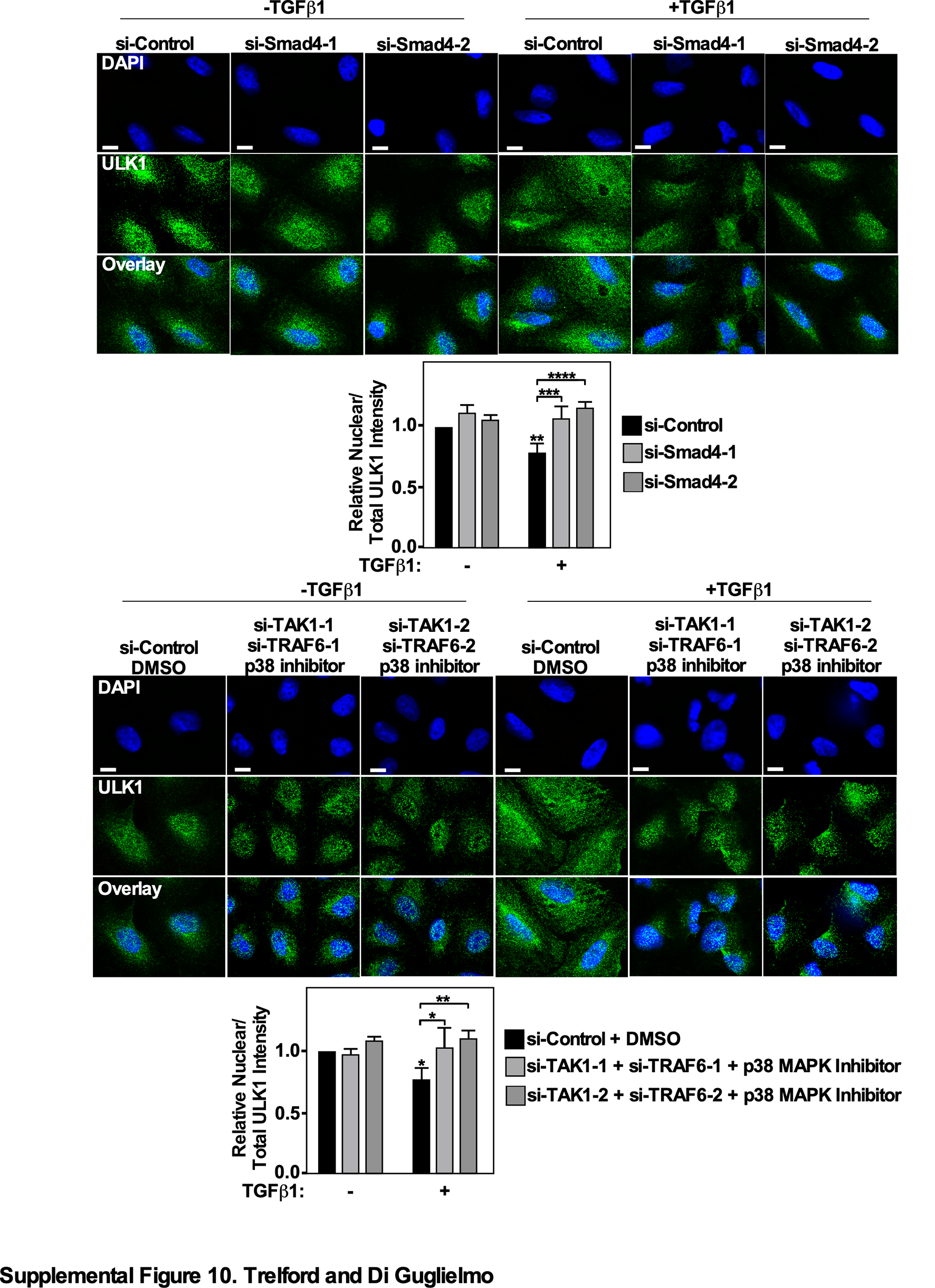

Supplement: Supplementary Figure 10 — The effect of canonical and non-canonical TGFβ signaling on the cellular distribution of ULK1. (A) A549 cells were transfected with si-Control or two different siRNAs targeting Smad4 (si-Smad4-1; s534708 and siSmad4-2; s8404) for 48 h. The cells were incubated in the absence or presence of 250 pM TGFβ1 for 24 h, fixed and stained with DAPI (blue) or anti-ULK1 (green). A Nikon Eclipse Ti2 confocal microscope was used to visualize the cells and an optical slice through the nucleus was imaged. ImageJ (version 2.0) quantified relative nuclear ULK1 intensity/Total ULK1 intensity, which are graphed below representative images (n = 3 ± SD). Significance is indicated as ∗∗ = P < 0.01, ∗∗∗ = P < 0.001, and **** = P < 0.0001. Bar = 10 μm. (B) A549 cells were transfected with si-Control or two different siRNAs against TAK1 (si-TAK1-1; s13766 and TAK1-2; s13767) or TRAF6 (si-TRAF6-1; s14388 and TRAF6-2; s14389) for 48 h. The cells were incubated in the absence or presence of 250 pM TGFβ1 and 10 μM P38 MAPK inhibitor for 24 h. The cells were then fixed and stained with DAPI (blue) or anti-ULK1 (green). A Nikon Eclipse Ti2 confocal microscope was used to visualize the cells and an optical slice through the nucleus was imaged. ImageJ (version 2.0) quantified relative nuclear ULK1 intensity/Total ULK1 intensity, which are graphed below representative images (n = 3 ± SD). Significance is indicated as ∗ = P < 0.05 and ∗∗ = P < 0.01. Bar = 10 μm. [file Image_10.TIF]
